# Exploration of the immune cell infiltration-related gene signature in the prognosis of melanoma

**DOI:** 10.18632/aging.202279

**Published:** 2021-01-10

**Authors:** Yangyang Zeng, Yulan Zeng, Hang Yin, Fengxia Chen, Qingqing Wang, Xiaoyan Yu, Yunfeng Zhou

**Affiliations:** 1 Hubei Key Laboratory of Tumor Biological Behaviors, Zhongnan Hospital of Wuhan University, Wuhan 430071, Hubei, China; 2 Department of Radiation and Medical Oncology, Zhongnan Hospital of Wuhan University, Wuhan 430071, Hubei, China; 3 Cancer Center, Union Hospital, Tongji Medical College, Huazhong University of Science and Technology, Wuhan 430022, Hubei, China

**Keywords:** melanoma, immune cell infiltration, prognostic signature, biomarkers

## Abstract

Melanoma is a life-threatening form of skin cancer with an elevated risk of metastasis and high mortality rates. The prognosis and clinical outcomes of cancer immunotherapy in melanoma patients are influenced by immune cell infiltration in the tumor microenvironment (TME) and the expression of genetic factors. Despite reports suggesting that immune-classification may have a better prediction of prognosis compared to the American Joint Committee on Cancer/Union for International Cancer Control (AJCC/UICC) TNM-classification, the definition of Immunoscore in melanoma is becoming a difficult challenge. In this study, we established and verified a 7-gene prognostic signature. Melanoma patients from the Cancer Genome Atlas (TCGA) were separated into a low-risk group and a high-risk group using the median risk score. Receiver operating characteristic (ROC) analysis for overall survival (OS) showed that the area under the curve (AUC) was 0.701 for 1 year, 0.726 for 3 years, and 0.745 for 5 years, respectively. Moreover, a nomogram was constructed as a practical prognostic tool, and the AUC was 0.829 for 3 years, and 0.803 for 5 years, respectively. Furthermore, we validated the above results in two datasets from the Gene Expression Omnibus (GEO) database and the relationship between 7-gene prognostic signature and immune infiltration estimated.

## INTRODUCTION

Melanoma is a highly aggressive skin cancer that causes about 55,500 deaths annually [1]. Metastatic melanoma represents the cause of death in the vast majority of cases and has a 5-year relative survival rate of approximately 25% [2, 3]. Based on clinical and pathological features, there are different therapeutic options for melanoma, including surgical treatment, chemotherapy, radiotherapy, immunotherapy, or targeted therapy [4, 5].

Previous studies have reported that the immune-classification may have a more superior prognostic value compared to the AJCC/UICC TNM-classification, which reveals the importance of immunological biomarkers in melanoma [6–9]. Despite the encouraging clinical outcomes of immunotherapy over the past decade, a significant proportion of patients develop resistance, which is a major obstacle to successful immunotherapy [3, 10]. In spite of considerable advances in genetic approaches that provide diagnostic, prognostic, or therapeutic information, the gene-based biomarkers have not yet been used in routine clinical practice [1, 11, 12]. Therefore, it is imperative to explore the immune-related genomic signature to supplement conventional prognostic factors and to improve the effects of immunotherapeutic drugs.

Increasing evidence shows that immune cell infiltration of the TME influences the prognosis and clinical outcome of cancer immunotherapy [13–16]. In melanoma, tumor-infiltrating lymphocytes are a favorable prognosticator and predictive biomarker for treatment response [17–19]. Moreover, increasing evidence suggests that an intense infiltration of B cells, activated T cells and mature dendritic cells could be associated with a positive prognosis, while an increased amount of macrophages, mast cells, and neutrophils predict worse survival in malignant melanoma [16, 20–22]. However, due to the difficulties experienced in the evaluation and the complex intratumoral immune reactions, the definition of Immunoscore is becoming a difficult challenge in melanoma [9, 23]. Although programmed death-ligand 1 (PD-L1) expression in tumor-infiltrating immune cells (TIICs) is related to a better prognosis of cancer, PD-L1 expression does not influence treatment decisions [1, 24]. Therefore, we identified potential immune-related prognostic genes for melanoma prognosis and therapeutic evaluation. A series of immune-related genes as potential prognostic predictors from TCGA was used to establish a 7-gene prognostic signature in melanoma. Furthermore, we validated the results in two GEO datasets and estimated the relationship between the established prognostic signature and immune infiltration.

## RESULTS

### Construction and verification of immune-related groups

**Figure 1:**
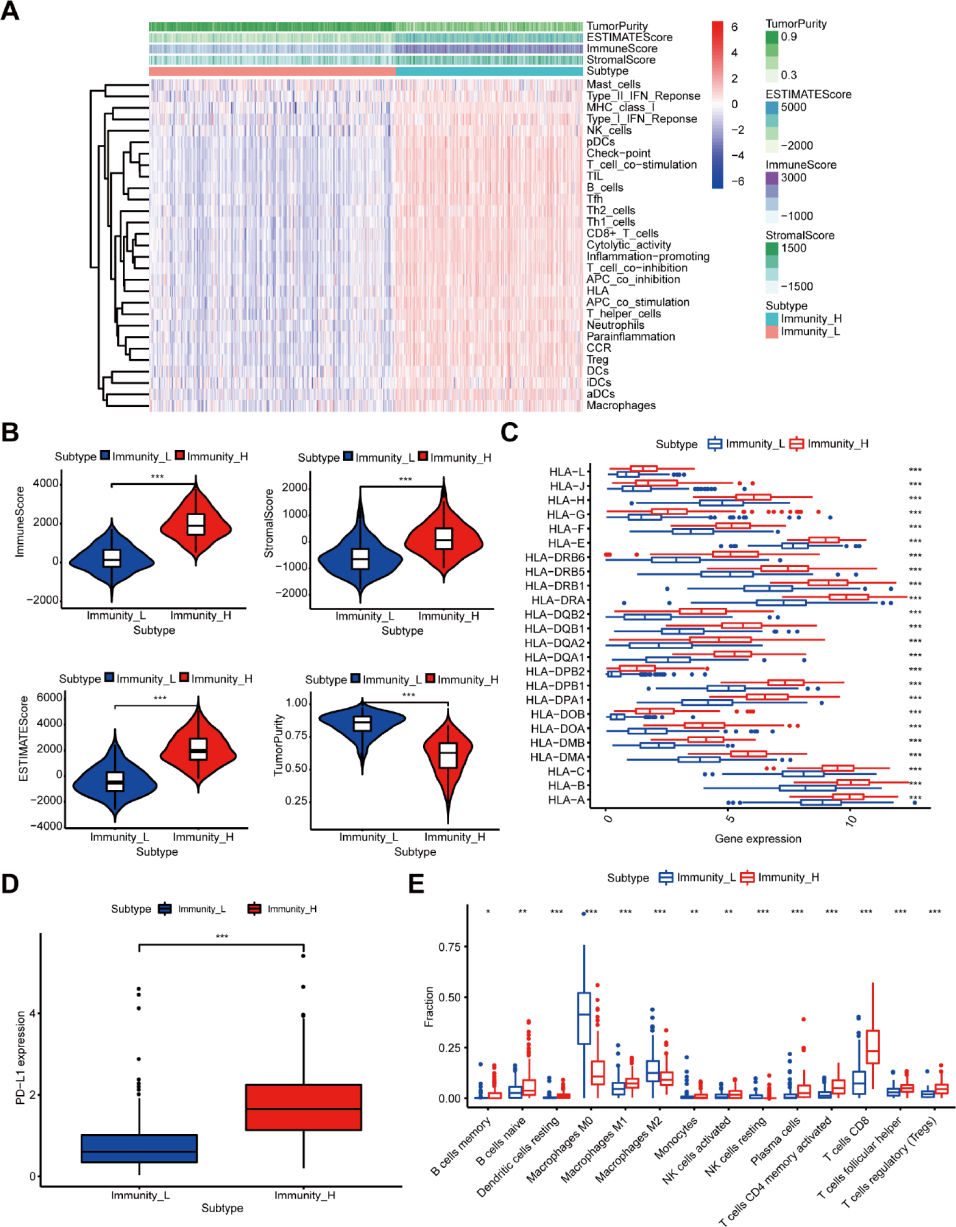
**Construction and verification of immune-related groups in melanoma samples.** (**A**) The high and low immune cell infiltration groups (Immunity_H and Immunity_L) were constructed by ssGSEA and further verified by the ESTIMATE algorithm. (**B**) The correlation between the scores calculated by the ESTIMATE algorithm and the two immune cell infiltration groups (P < 0.001). (**C**) The expression of HLA family genes. (**D**) The expression of PD-L1. (**E**) The difference of TIIC subtypes between the two immune cell infiltration groups.

A total of 471 melanoma samples from the TCGA dataset were classified into two immune-related groups using the single-sample gene set enrichment analysis (ssGSEA): 203 samples (43.1%) in the high immune cell infiltration group, and 268 samples (56.9%) in the low immune cell infiltration group (Figure 1A).

Moreover, we verified the reliability of the two immune-related groups classified by the above method. Firstly, we compared the degree of immune cell infiltration between immune-related groups by the Estimation of Stromal and Immune cells in Malignant Tumor tissues using the Expression data (ESTIMATE) algorithm. As expected, the Tumor Purity was higher in the low immune cell infiltration group, while the ESTIMATE Score, Immune Score, and Stromal Score were higher in the high immune cell infiltration group (Figure 1A). Figure 1B shows the significant associations between the scores and the two immune-related groups (P < 0.001). We further found significantly higher expression level of the human leukocyte antigen (HLA) family genes and PD-L1 (Figure 1C, 1D, P < 0.001) and increased variety of immune cells among 22 TIICs subtypes in the high immune cell infiltration group compared with the other group (Figure 1E).

Furthermore, the results of gene set enrichment analysis (GSEA) suggested an intensive immune phenotype in the high immune cell infiltration group. The top 5 Gene ontology (GO) terms are shown in Supplementary Figure 1A, including the MHC class II protein complex and immunoglobulin complex. Moreover, 5 immune-related Kyoto Encyclopedia of Genes and Genomes (KEGG) pathways were selected and shown in Supplementary Figure 1B, such as B cell and T cell receptor signaling pathway. As previously reported, we also confirmed that the immune cell infiltration was a favorable prognosticator in melanoma (Supplementary Figure 2), and was closely associated with the above results. Therefore, our immune cell infiltration groups could be used as immune-related groups for further analysis.

### Screening of differentially expressed genes (DEGs) and functional analysis

**Figure 2:**
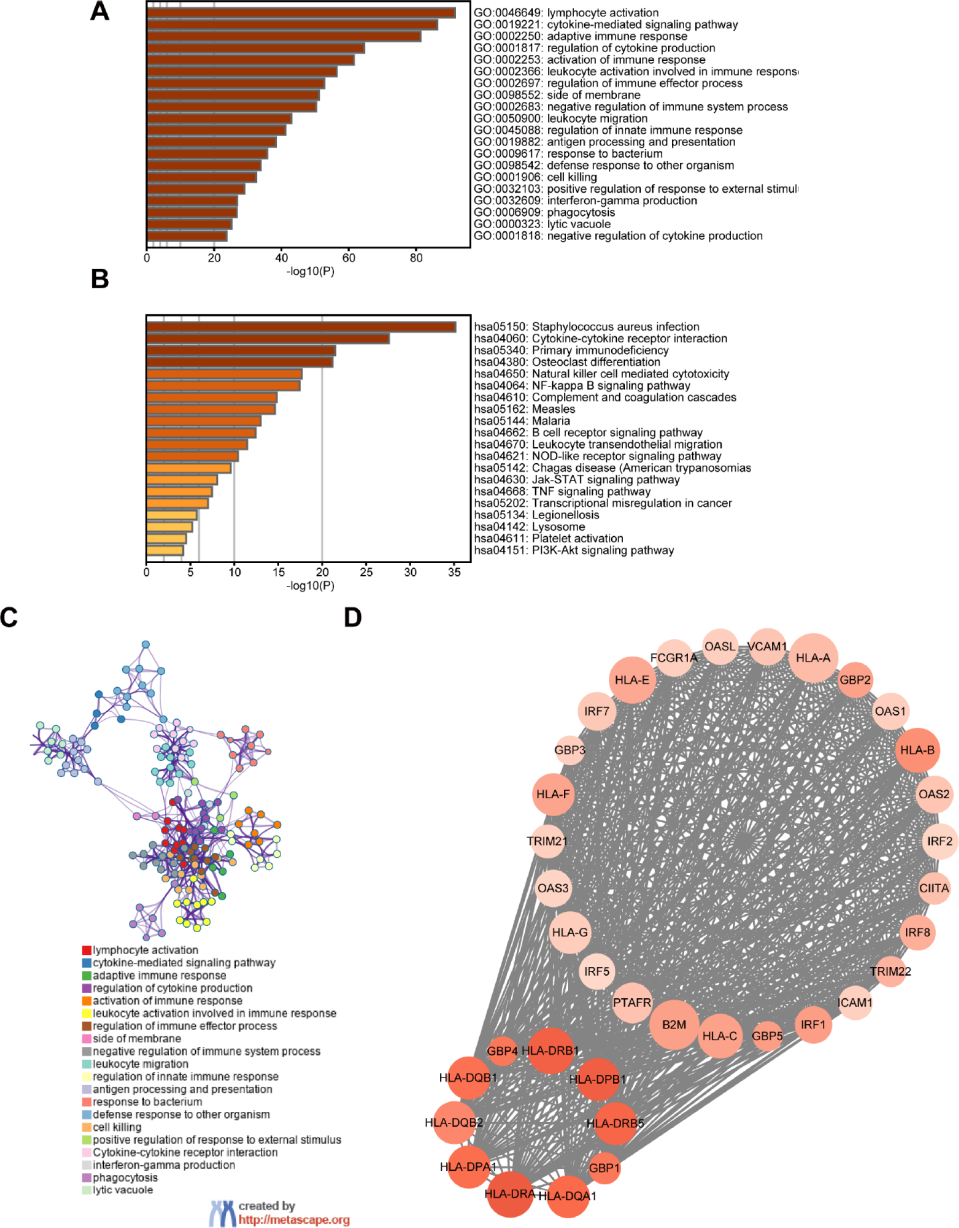
**Screening of DEGs and functional analysis.** (**A**) Heatmap of the GO enriched terms. (**B**) Heatmap of the KEGG enriched terms. (**C**) A network of GO and KEGG enriched terms. (**D**) The top one module. The color of nodes represents the log (FC) value and the size reflects the number of interacting proteins with the designated protein.

A total of 659 DEGs were identified from a comparison of high vs. low immune cell infiltration groups in the TCGA dataset, and comprised of 607 up-regulated genes and 52 down-regulated genes. Functional analysis was performed using Metascape. The top GO terms and KEGG included lymphocyte activation and cytokine-cytokine receptor interaction (Figure 2A, 2B). The network of GO and KEGG enriched terms is shown in Figure 2C. Moreover, the 659 DEGs were analyzed using the Search Tool for the Retrieval of Interacting Genes database (STRING) and were used to construct the protein-protein interaction (PPI) network. Several central modules were established by Molecular Complex Detection (MCODE) software (Figure 2D, Module 1). Furthermore, we determined the prognostic value of the 659 DEGs in TCGA and obtained 509 DEGs associated with OS (Supplementary Table 1).

### Identification of immunity-related module and intersecting DEGs

**Figure 3:**
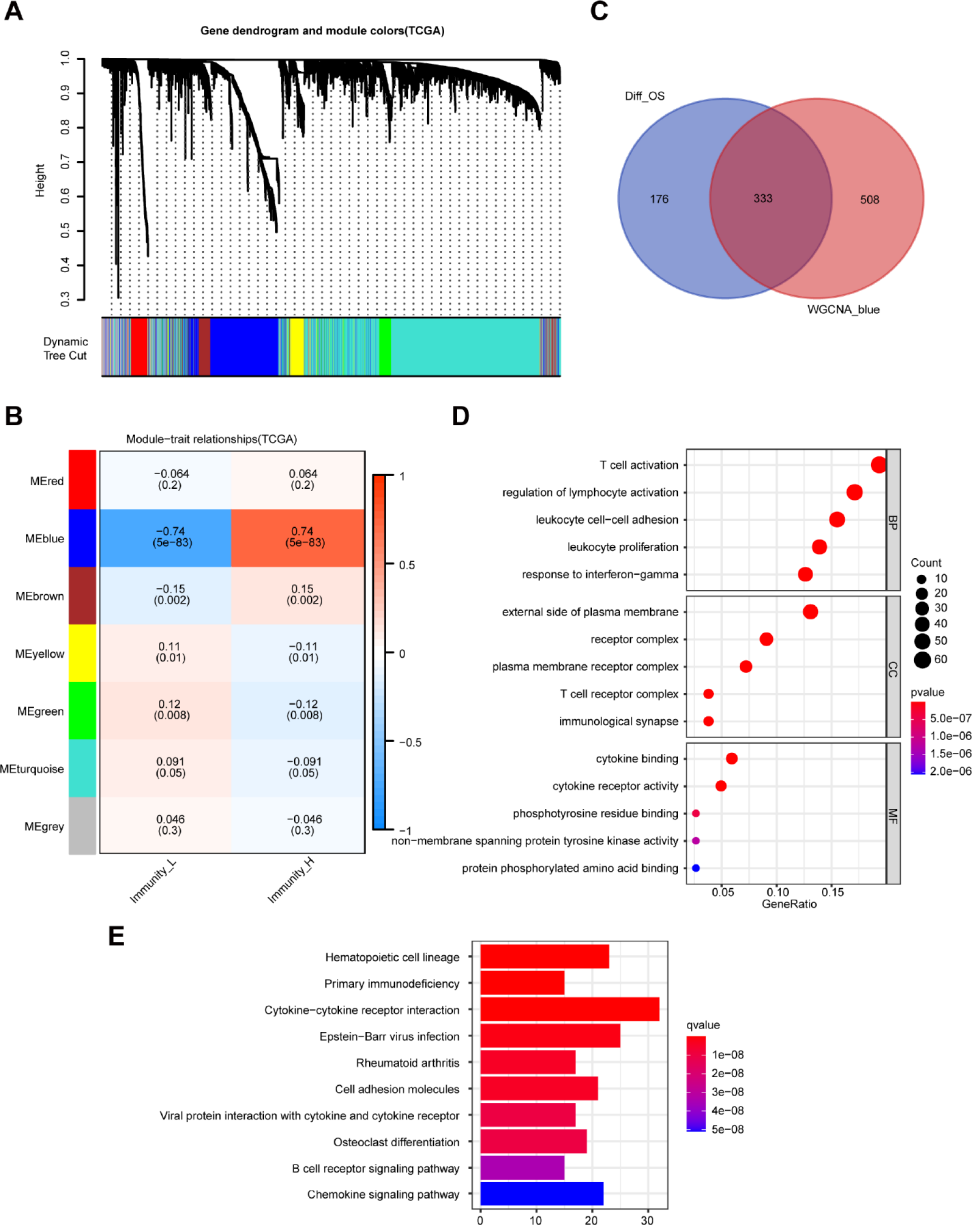
**Identification of highly immune-related DEGs with prognostic value and functional analysis.** (**A**) A hierarchical clustering dendrogram is built to detect co-expressed genes in modules in the TCGA dataset of melanoma. (**B**) A heatmap showing the relationships of consensus module-trait in different modules under the low and high immune cell infiltration groups. (**C**) Identification of 333 common DEGs from 509 DEGs with prognostic value and the blue module using the Venn diagram software. (**D**) Top 15 GO terms. (**E**) Top 10 KEGG pathways.

The weighted gene co-expression network analysis (WGCNA) detected 7 co-expression modules and their association with immune-related groups were analyzed. The cluster dendrogram was established (Figure 3A) and the blue module was the most correlated module of the immune cell infiltration groups (|r| = 0.74, P = 5e-83) (Figure 3B). A total of 841 genes in the selected module were extracted for further study (Supplementary Table 2), and 333 intersecting DEGs were obtained from the module with the highest correlation coefficient and 509 DEGs with prognostic value (Figure 3C).

Functional analysis of the 333 DEGs was carried out by the clusterProfiler package. Top GO terms included T cell activation in biological processes (BP), T cell receptor complex in cellular components (CC), and cytokine binding in molecular functions (MF) (Figure 3D). Moreover, the top 10 enriched pathways are shown in Figure 3E, including cytokine-cytokine receptor interaction and B cell receptor signaling pathway.

### Establishment and validation of the 7-gene prognostic signature

**Figure 4:**
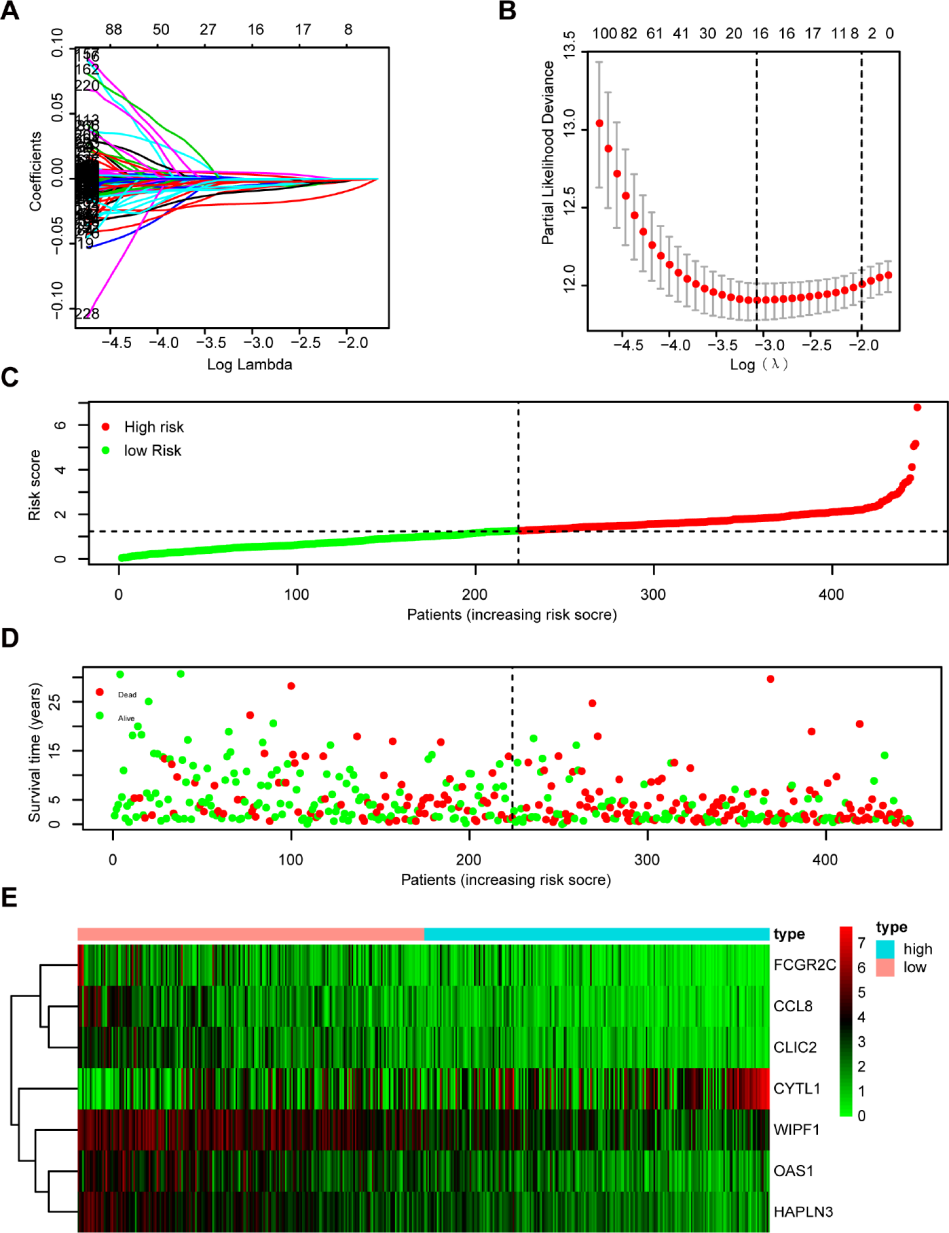
**Establishment and assessment of immune-related prognostic signature.** (**A**, **B**) 294 DGEs determined using (**A**) LASSO regression analysis and (**B**) 10-fold cross-validation. (**C**–**E**) The distribution of (**C**) risk score, (**D**) survival status, and (**E**) immune-related gene expression profiles.

The 333 DEGs were subjected to univariate Cox regression analysis and 294 DEGs were selected (P < 0.05) for further study. Using the Least Absolute Shrinkage and Selection Operator (Lasso) Cox regression analysis, 16 genes were filtered for stepwise multivariate Cox regression analysis (Figure 4A, 4B). Finally, 7 key genes (CYTL1, CCL8, FCGR2C, OAS1, HAPLN3, WIPF1, CLIC2) were used to construct the prognostic signature (Supplementary Figure 3): Risk score = 0.007* CYTL1 - 0.033* CCL8 - 0.021* FCGR2C - 0.015* OAS1 - 0.037* HAPLN3 - 0.014* WIPF1 - 0.023* CLIC2 (Supplementary Table 3). The distribution of the risk score, survival status, and gene expression profiles between the two groups are displayed in Figure 4C–4E.

**Figure 5:**
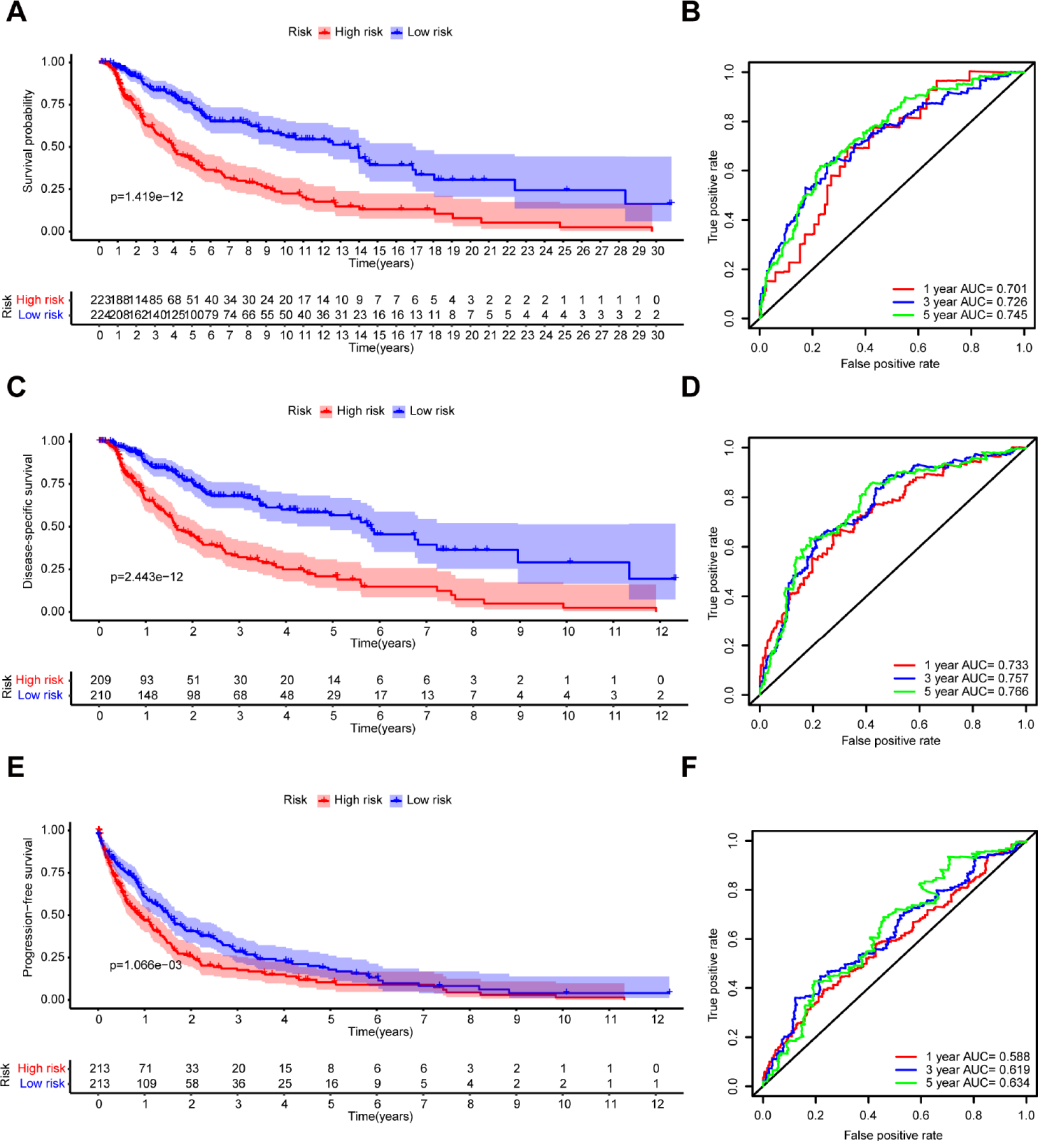
**Kaplan–Meier survival analysis and time-dependent ROC analysis of the 7-gene prognostic signature in the TCGA dataset.** Kaplan-Meier survival curves and ROC curves of (**A**, **B**) OS, (**C**, **D**) DSS, and (**E**, **F**) PFS in patients with melanoma.

Melanoma patients from TCGA were separated into a low-risk group (n = 224) and a high-risk group (n = 223) based on the median risk score as a cutoff value. The Kaplan-Meier analysis showed that the high-risk group had a poorer OS compared with the low-risk group (Figure 5A). Moreover, the time-dependent ROC analysis showed that the AUC was 0.701 for 1 year, 0.726 for 3 years, and 0.745 for 5 years, respectively, indicating that our prognostic signature had a strong predictive capacity (Figure 5B). Similar results were observed for DSS and PFS. The high-risk group had worse DSS (Figure 5C) and PFS (Figure 5E) compared with the low-risk group. The AUCs for 1-year, 3-year, and 5-year DSS rates using the 7-gene prognostic signature were 0.733, 0.757, and 0.766, respectively (Figure 5D). The AUCs for predicting 1-year, 3-year, and 5-year PFS were 0.588, 0.619, and 0.634, respectively (Figure 5F). These results demonstrated that the 7-gene prognostic signature was an effective prognostic indicator of OS, DSS, and PFS.

Furthermore, GSE54467 (n = 79) and GSE65904 (n = 207) were utilized to validate the predictive value of OS and DSS. Based on the median risk score, the high-risk group (n=39) and low-risk group (n=40) were identified in the GSE54467 dataset. Likewise, patients in the high-risk group had a shorter OS (Supplementary Figure 4A). The AUC of our prognostic signature was 0.555 at 1 year, 0.682 at 3 years, and 0.761 at 5 years (Supplementary Figure 4B). In the GSE65904 dataset, the Kaplan-Meier analysis also showed that the high-risk group (n=103) had a worse DSS compared with the low-risk group (n=104) (Supplementary Figure 4C). The time-dependent ROC analysis also showed that our prognostic signature had a good predictive capacity in predicting 1-year, 3-year, and 5-year DSS (AUC = 0.641, 0.629, 0.590, respectively) (Supplementary Figure 4D).

**Figure 6:**
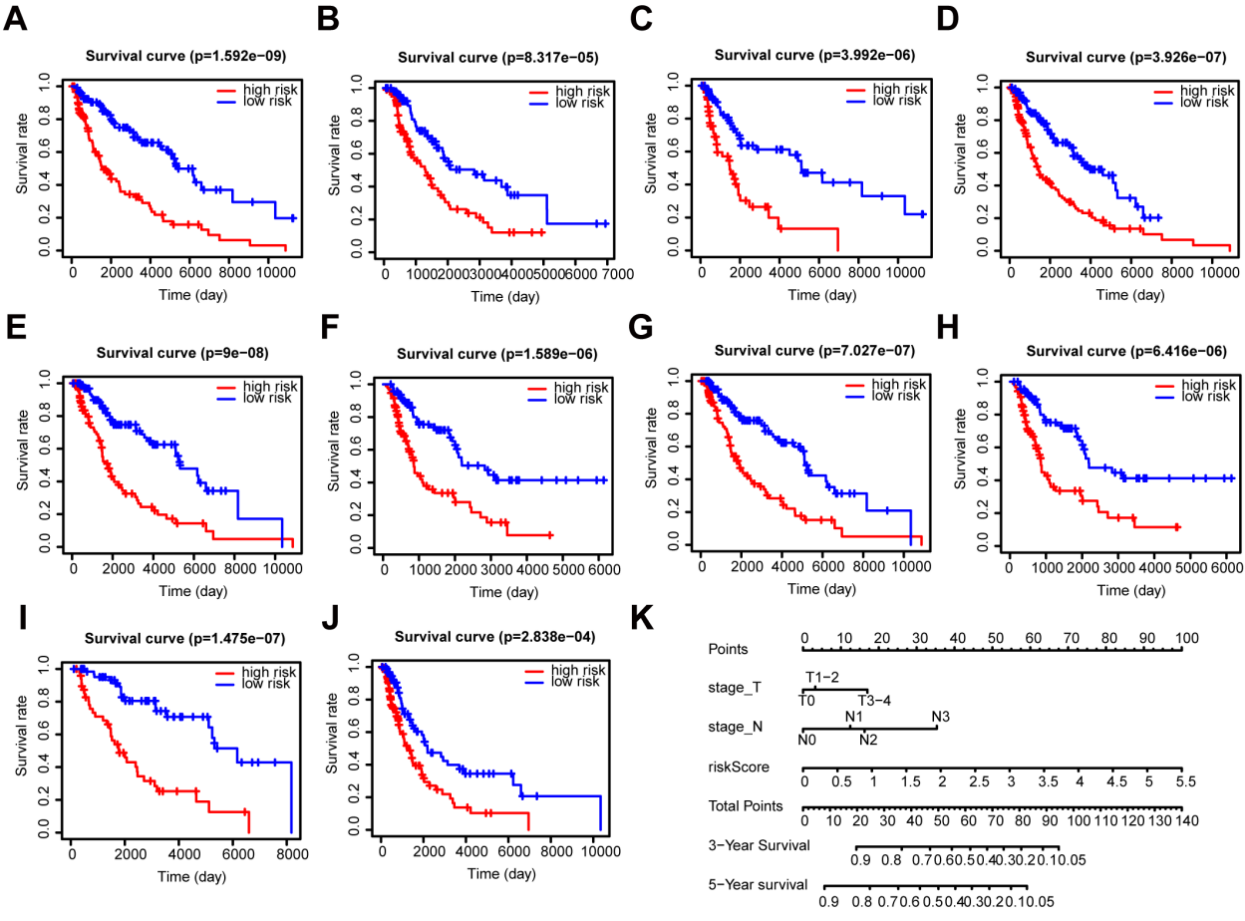
**Stratification analysis and construction of a prognostic nomogram in patients.** (**A**–**J**) Kaplan–Meier analysis of the subgroups including (**A**) ≤ 60 years, (**B**) > 60 years, (**C**) female, (**D**) male, (**E**) stage I-II, (**F**) stage III-IV, (**G**) N0, (**H**) N1-3, (**I**) T1-2, and (**J**) T3-4. (**K**) OS predictive nomogram.

Further, we performed a stratification analysis for OS to determine the prognostic power of our prognostic signature in subgroups of melanoma. The high-risk patients had a worse prognosis compared with the low-risk patients in each stratum (Figure 6A–6J). Moreover, the prognostic signature had a better predictive ability compared with other clinical factors (Supplementary Figure 5A–5C). The pathologic T stage, N stage, and risk score were identified as independent prognostic factors (P < 0.001) (Supplementary Figure 5D, 5E). Eventually, we incorporated these prognostic factors to formulate an OS nomogram (Figure 6K). The ROC curves and calibration curves showed a favorable predictive capacity and stability in predicting the 3-year (AUC = 0.829) or 5-year (AUC = 0.803) OS (Supplementary Figure 6).

### Immune infiltration score and immune cells infiltration analysis

**Figure 7:**
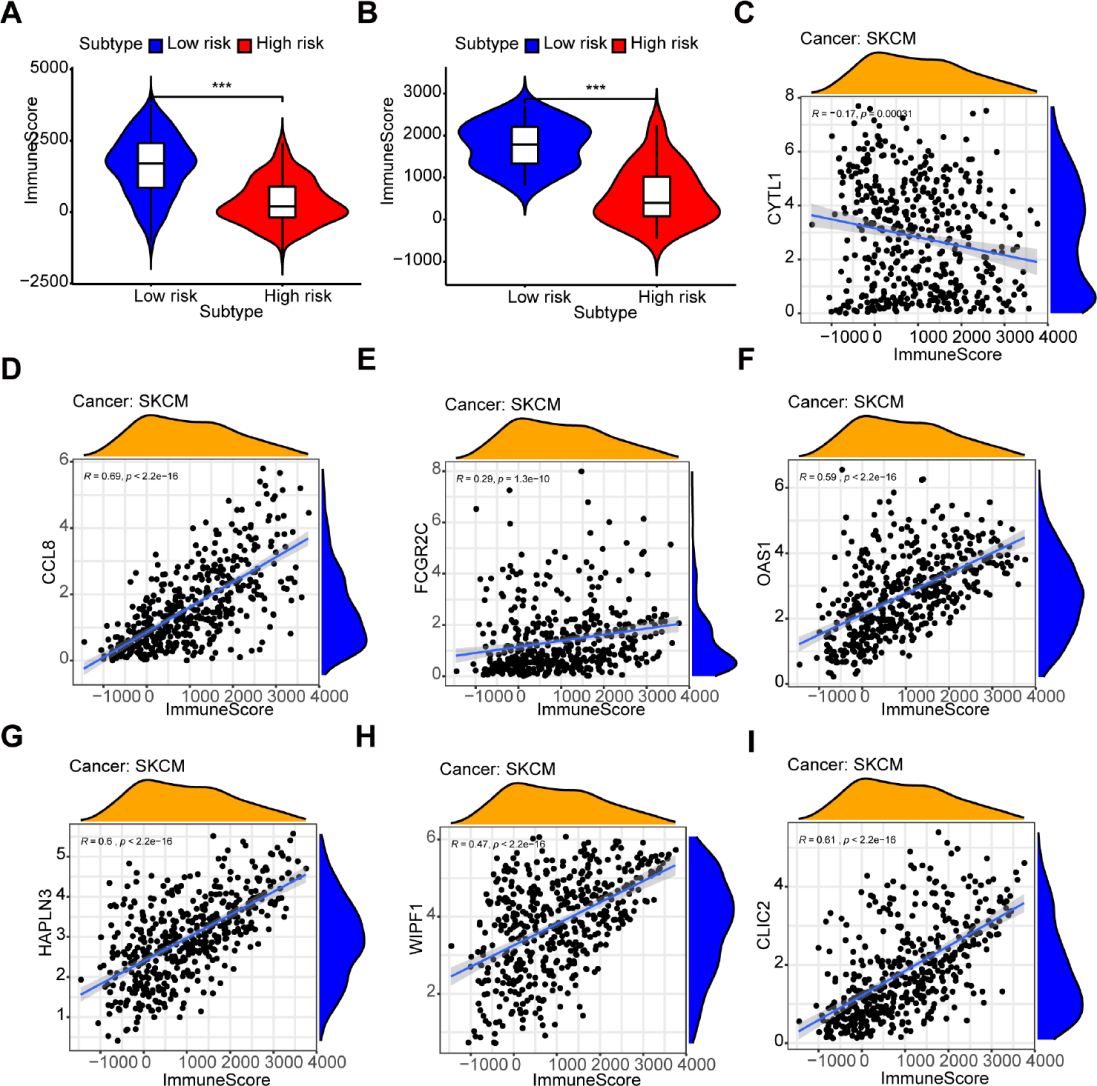
**Relationship between the prognostic signature and immune score.** (**A**, **B**) The high-risk group has lower immune scores compared with the low-risk group in (**A**) the TCGA dataset and (**B**) GSE54467. (**C**–**I**) The association between immune score and the expression of each gene in a 7-gene prognostic signature: (**C**) CYTL1, (**D**) CCL8, (**E**) FCGR2C, (**F**) OAS1, (**G**) HAPLN3, (**H**) WIPF1, (**I**) CLIC2.

The box chart showed that there were higher immune and stromal scores in the high-risk group compared with the low-risk group (Figure 7A and Supplementary Figure 7A, P < 0.001). The results in GSE54467, an independent dataset, also confirmed a similar correlation (Figure 7B and Supplementary Figure 7B, P < 0.001). Additionally, the expression of each gene (CCL8, FCGR2C, OAS1, HAPLN3, WIPF1, CLIC2) was significantly positively correlated to the immune score (Figure 7D–7I) and stromal score (Supplementary Figure 7D–7I). But CYTL1 had the opposite result in the immune score (Figure 7C) and had no relevance to the stromal score (Supplementary Figure 7C).

**Figure 8:**
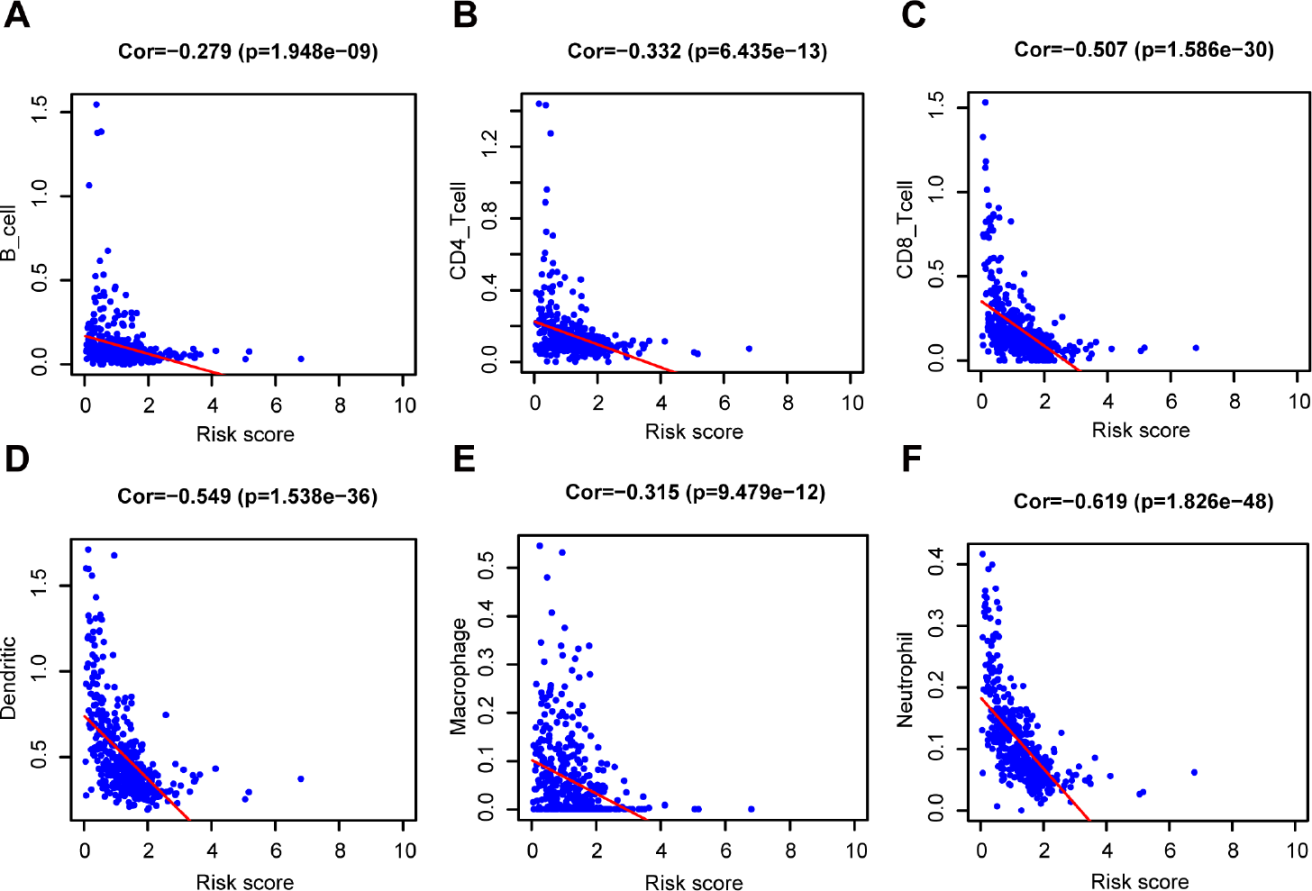
**Relationship between the prognostic signature and the TIICs subtypes.** Association between risk score and six subtypes of TIICs: (**A**) B cells. (**B**) CD4+ T cell. (**C**) CD8+ T cell. (**D**) Dendritic cells. (**E**) Macrophage. (**F**) Neutrophil.

Furthermore, the risk score was significantly negatively correlated to B cells (Cor = -0.279), CD4+ T cells (Cor = - 0.332), CD8+ T cells (Cor = - 0.507), dendritic cells (Cor = - 0.549), macrophages (Cor = - 0.315), and neutrophils (Cor = - 0.619) (Figure 8, P < 0.001). Meanwhile, 6 prognostic genes (CCL8, FCGR2C, OAS1, HAPLN3, WIPF1, CLIC2) were associated with the abundance of B cell, CD4+T cell, CD8+T cell, dendritic cells, neutrophils and macrophages (Supplementary Figure 8B–8G, P < 0.05). The only gene (CYTL1) was unrelated to the 6 types of immune cell infiltration (Supplementary Figure 8A, P > 0.05).

### GSEA analysis

**Figure 9:**
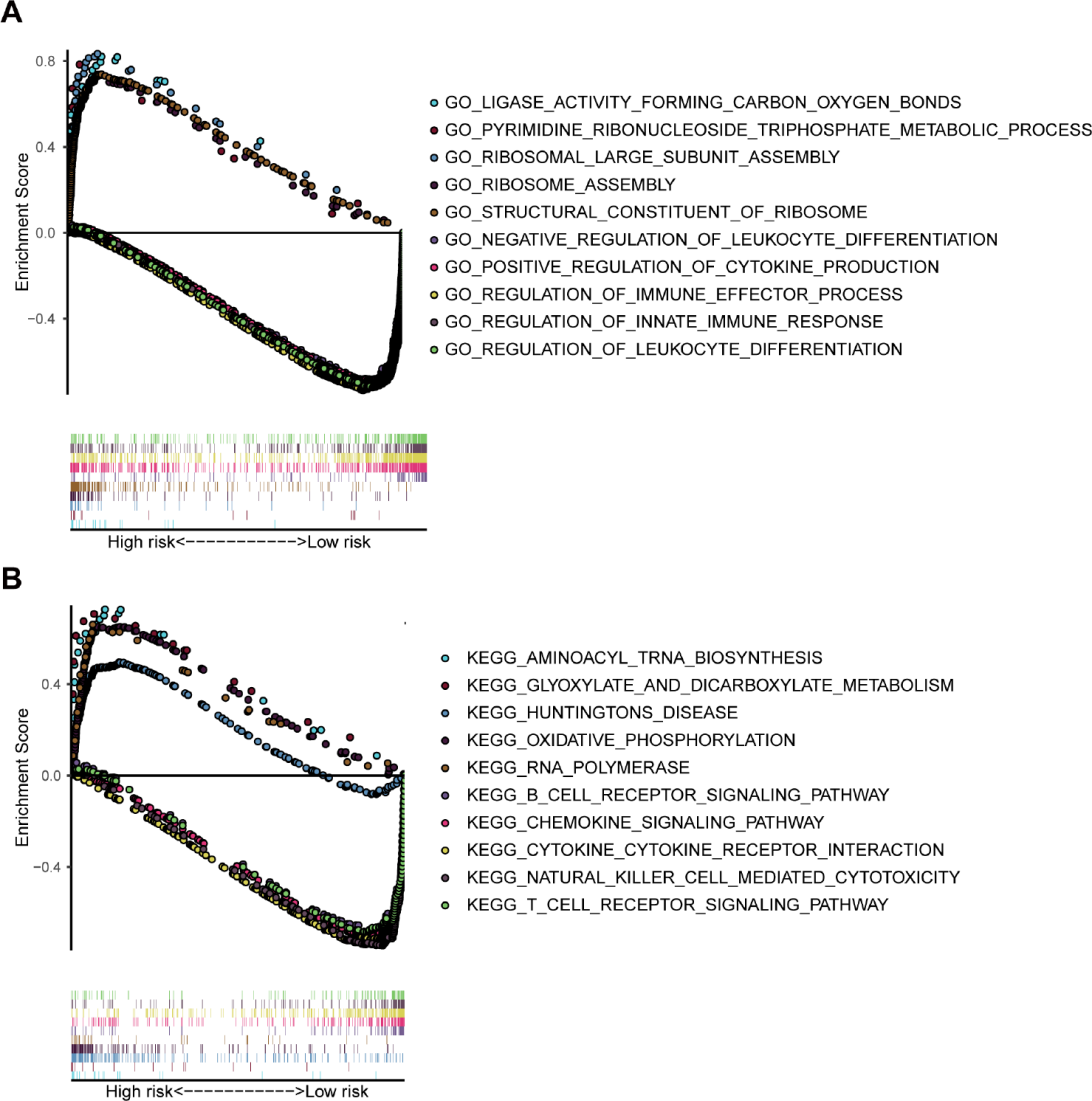
**GSEA analysis based on the risk value.** (**A**) The significant enrichment of the top 5 GO terms in the high-risk group and the top 5 immune-related GO terms in the low-risk group. (**B**) The significant enrichment of the top 5 pathways in the high-risk group and the top 5 immune-related pathways in the low-risk group.

The GSEA analysis was performed for functional annotation of the 7-gene prognostic signature. We found that more than 10 immune-related GO terms and KEGG pathways were visibly enriched in the low-risk group, while there were few enriched in the high-risk group. The top 5 immune-related GO terms are shown in Figure 9A, such as positive regulation of cytokine production. Besides, the top 5 immune-related pathways are displayed in Figure 9B, such as natural killer cell mediated cytotoxicity.

## DISCUSSION

**Figure 10:**
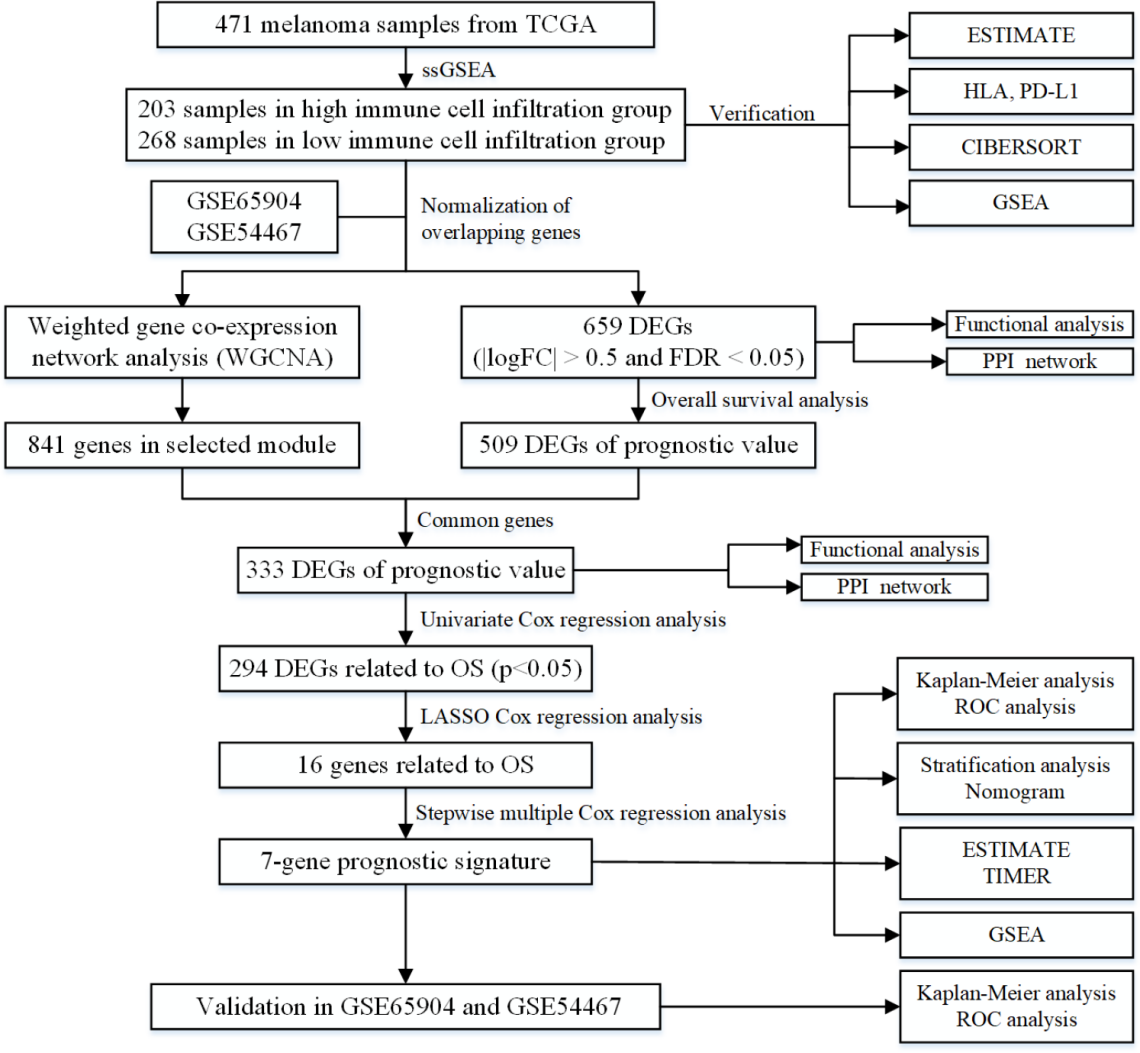
**The flowchart of the current study.**

In this work, two immune-related groups were classified based on the degree of immune cell infiltration in the TCGA dataset of melanoma. A total of 333 DEGs with prognostic value were obtained by WGCNA. A novel 7-gene prognostic signature in melanoma was established and its predictive value and accuracy validated in GSE54467 and GSE65904 datasets. Finally, the interaction between the immune and 7-gene prognostic signature was explored using the ESTIMATE algorithm, TIMER database and GSEA. Figure 10 displays a detailed flowchart of the current study.

Although studies have reported that the immune-classification may have a better prognostic value than the AJCC/UICC TNM-classification, the definition of Immunoscore is a challenge in melanoma [23]. Previous studies suggest that the immune infiltration of the TME can be used to evaluate melanoma survival [25–27]. Therefore, we identified potential immune-related prognostic genes for melanoma prognosis. In our study, we established immune-related groups by ssGSEA and identified them using the ESTIMATE algorithm. We verified the reliability of the two immune-related groups by comparing different expressions of HLA family genes and PD-L1, which influence the human immune system and immunotherapy [28, 29], and using the CIBERSORT algorithm. Furthermore, the results of GSEA also showed different immune phenotypes in immune-related groups.

Based on the high heterogeneity of melanoma, there are more than 51,000 biomarkers, including tissue- based tumor cell and TME biomarkers [30, 31]. In this study, we obtained 659 DEGs, and 333 DEGs with prognostic value were identified and validated in the TCGA dataset by WGCNA. Functional analysis was performed and PPI networks for these genes were constructed. The results of the GO analysis in both 659 DEGs and 333 DEGs were closely related to the TME and immune function, such as lymphocyte activation and T cell activation. Besides, KEGG analysis showed that the B cell receptor signaling pathway was the overlapping enriched pathways. In the top one PPI module of the 659 DEGs, the main hub nodes were reported to affect the TME and immunotherapy, including HLA and guanine-binding protein (GBP) family induced by interferon and C-X-C motif chemokine ligand, like HLA-A [32] and GBP4 [33].

In this study, 7 genes (CYTL1, CCL8, FCGR2C, OAS1, HAPLN3, WIPF1, CLIC2) were identified as prognostic signatures for melanoma. CYTL1 was found to be highly expressed in neuroblastoma and a potential therapeutic target and diagnosis biomarker [34]. The expression of CCL8 in nude mice has been reported to inhibit human cervical cancer [35]. Moreover, in melanoma, previous research suggests that a local CCL8-rich environment promotes the selection of metastatic tumor cells, while a high CCL8 concentration inhibits their migration [36]. OAS1 induced by interferons is reported to be one gene in prognostic signature for patients with bladder cancer, despite a lack of sufficient experimental studies [37]. A previous study showed that WIPF1 knockdown inhibited natural killer cell cytotoxicity [38, 39]. CLIC2 is identified as a novel gene related to immune checkpoint proteins based on TCGA gene expression data [40], while another study suggests that it inhibits the hematogenous spread of tumor cells [41]. However, there is a need for further research to understand the function of FCGR2C and HAPLN3 in cancer. The role of the 7 genes in melanoma also requires more in-depth investigation.

Additionally, our prognostic signature was found to be an effective and stable prognostic indicator, since it demonstrated favorable prognostic value in predicting OS, DSS, and PFS outcomes in the TCGA dataset of melanoma. Two GEO datasets were used to validate the OS and DSS results. Moreover, stratification analysis in different subgroups was performed and a nomogram was constructed by integrating each independent clinical prognostic factor. Both ROC curves and calibration plots showed a robust and reliable predictive ability of the nomogram for OS. Therefore, our prognostic signature could predict more clinical prognostic factors and provide a useful tool to supplement the traditional clinical prognostic factors and improve the therapeutic effect. Nevertheless, the limitations of this study included that some risk factors for melanoma like a family history of melanoma could not be collected in the TCGA dataset, and the two selected GEO datasets lacked some corresponding clinical features data.

As for the interaction between the immune and the established 7-gene prognostic signature, we found that the low-risk group had higher immune and stromal scores. Previous studies demonstrated that intense infiltration of activated T cells, B cells, or mature dendritic cells could be positive prognosis factors [16, 20, 21]. Infiltrating CD8 lymphocytes are also reported to be a good prognostic factor for melanoma [42]. We observed a significantly negative correlation between the abundance of six immune cell subtypes and the risk score. Initially, neutrophils showed the strongest negative correlation with the risk score. However, it could be explained that the prognostic signature is mainly related to T cell activity and cytokine function: on one hand, CD8+ plays a major anti-tumor function, while on the other hand, some related cytokines may recruit anti-neoplastic neutrophils in the early stage [43]. Besides, the proportion of various immune infiltrating cells need to be considered for specific risk grouping. Moreover, except for CYTL1, the expression of each gene in the 7-gene prognostic signature was also found to be significantly related to immunization, such as immune score, stromal score and immune cell subtypes. Finally, GSEA analysis showed that many immune-related GO terms and KEGG pathways were enriched in the low-risk group. Due to the limitations associated with many current experiments on finite cell lines or samples, we carried out a comprehensive and systematical analysis of the TME in melanoma using a larger volume of data. However, despite the high heterogeneity of cancer or the lack of computer algorithms, continued exploration of these findings are likely to lead to novel insights into the molecular mechanisms.

In conclusion, this study identified and validated 333 immune infiltration related genes as potential immune predictors for melanoma. Furthermore, we constructed and verified a 7-gene prognostic signature associated with the TME as a prognostic indicator. Although previous studies have studied immune infiltration-related prognostic models, especially those on melanoma [25, 26, 44, 45], in contrast, we first established reliable immune-related groups and obtained core immune infiltration related genes with prognostic value. We then constructed a favorable prognostic model for use in predicting more clinical prognostic factors and established a nomogram as a practical prognostic tool. Finally, we explored the relationship between the model, including its genes, immune infiltration score, and immune cell infiltration. Taken together, this study provides a series of novel immune-related biomarkers for prognosis of melanoma and establishes a 7-gene prognostic signature to supplement traditional clinical prognostic factors and improve the therapeutic effect.

## MATERIALS AND METHODS

### Database

The HTSeq-FPKM data and clinical information of melanoma were acquired from the TCGA database (https://portal.gdc.cancer.gov). The cohort of melanoma for identifying immune-related genes consisted of 471 samples in TCGA. DSS and PFS data for melanoma was obtained from cBioportal (https://www.cbioportal.org/). Two GEO datasets (GSE54467, GSE65904) were gained from the GEO database (https://www.ncbi.nlm.nih.gov/geo/). Only patients with overall survival days ≥ 30 days were included in the study.

### Construction of immune-related groups

The infiltration levels of immune cells in 471 melanoma samples were estimated by ssGSEA [46], and the immune-related groups were constructed using the hclust function in R software. The ssGSEA analysis was carried out in the R package Gene Set Variation Analysis (GSVA) [47].

### Verification of immune-related groups

The related scores for individual samples were determined by the ESTIMATE algorithm in R software. Then, the different gene expression levels of the HLA family genes and PD-L1 were analyzed between the immune-related groups. Moreover, the composition of 22 immune cell subtypes was determined by the CIBERSORT package [48]. The immune-related functional annotation and signaling pathways that were differentially activated in the two groups were validated by GSEA.

### Screening of differentially expressed genes

The overlapping genes and normalized gene expression profile data in the TCGA dataset and the two GEO datasets were identified for further analysis. The comparison of high vs. low immune cell infiltration groups was executed in R package limma [49]. |logFC| > 0.5 and false discovery rate (FDR) < 0.05 were considered as the cut-off criteria.

### Functional analysis

Functional analysis was carried out by Metascape (http://metascape.org) [50] or R package clusterProfiler. The PPI network with the confidence score > 0.9 was established using the STRING website (https://string-db.org/) [51] and Cytoscape software [52], and proteins with degree > 1 were selected.

### Survival analysis

The Kaplan-Meier survival analysis was carried out to evaluate the correlation between the survival probability of patients and different groups characterized by different levels of immune cell infiltration, expression of individual gene, or risk score.

### WGCNA of immune-related groups

The WGCNA was used to determine the relationship between co-expression gene modules and immune-related groups in the WGCNA package [53]. The WGCNA network and the co-expressed gene modules were established and detected using the soft threshold power of β = 5, topological overlap matrix (TOM), and minimal module size of 50. 841 genes in the blue module with the highest correlation coefficient were selected for further analyses. A total of 333 DEGs with prognostic value in the TCGA dataset were obtained using the Venn diagram software.

### Identification of immune-related gene prognostic signature

A total of 294 DEGs (P < 0.05) were selected by univariate Cox regression analysis. Out of these, 16 genes were identified by LASSO Cox analysis with 10-round cross-validation. The prognostic signature was selected and optimized by stepwise multiple Cox regression analysis. The formula for the risk score was as follows [54]: Risk core = $\sum_{i=1}^{n}{\text{Coef}}_{i}{\text{*Exp}}_{i};$ *n*, Coef_*i*_, Exp_*i*_ represented the number of signature genes, the coefficient, and the gene expression level, respectively. Univariate and multivariate Cox regression analysis were performed to evaluate the independence of the prognostic signature from clinical factors. The time-dependent ROC analysis was performed using the survival package. The nomogram and calibration curves were plotted by R package rms.

### TIMER database analysis

The deconvolution algorithm provided by Tumor Immune Estimation Resource (TIMER) database (https://cistrome.shinyapps.io/timer/) [55] was used to determine the relationship between risk scores and TIICs using Pearson correlation. Additionally, the association between the expression of 7 genes and TIICs was also analyzed.

### Statistical analysis

Statistical analyses were carried out in R version 3.6.3 (Package: limma, sva, pheatmap, ggpubr, org.Hs.eg.db, clusterProfiler, enrichplot, survival, glmnet, survminer, survivalROC, beeswarm, rms, etc).

## Supplementary Material

Supplementary Figures

Supplementary Table 1

Supplementary Table 2

Supplementary Table 3
