# Supplementary material for: Exploration of the immune cell infiltration-related gene signature in the prognosis of melanoma
Source: Aging (Albany NY). 2021 Jan 10;13(3):3459–82. doi: 10.18632/aging.202279 (PMC7906183; doi:10.18632/aging.202279)
Supplement: Supplementary Figures [file aging-13-202279-s001.pdf]

SUPPLEMENTARY FIGURES

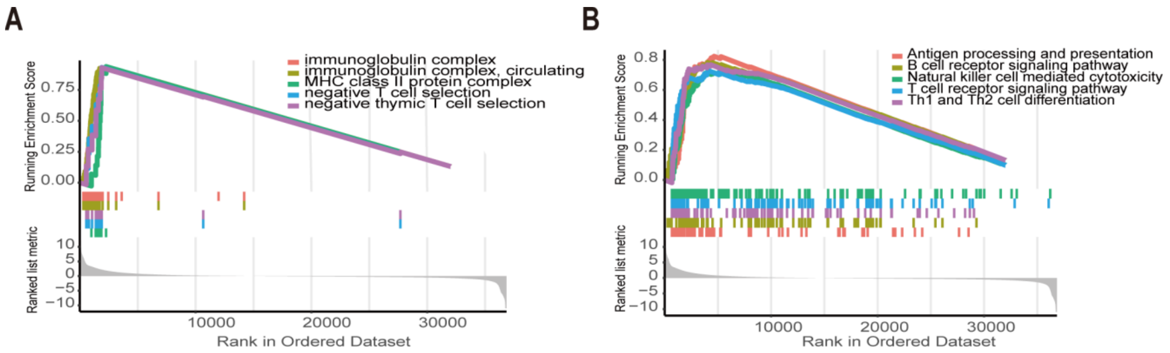

Supplementary Figure 1. GSEA analysis comparing the immune phenotype between the two immune-related groups. (**A**, **B**) In high immune cell infiltration group, (**A**) the significant enrichment of top 5 GO terms, and (**B**) the significant enrichment of 5 immune-related KEGG pathways.

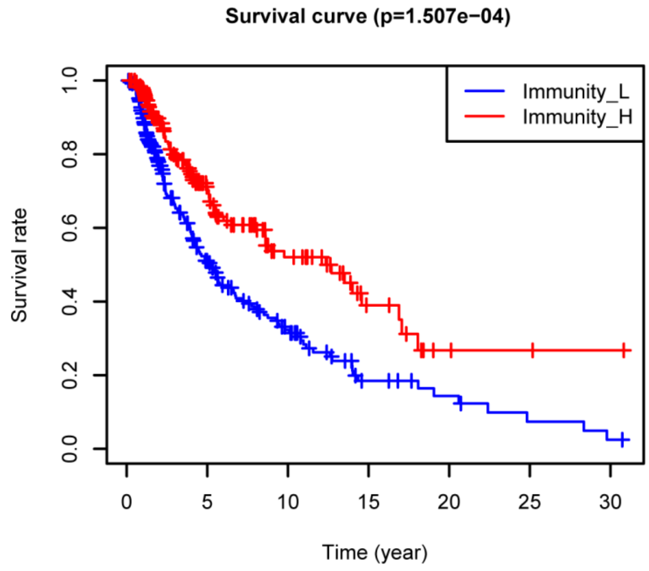

Supplementary Figure 2. Association between two immune-related groups and OS for melanoma patients.

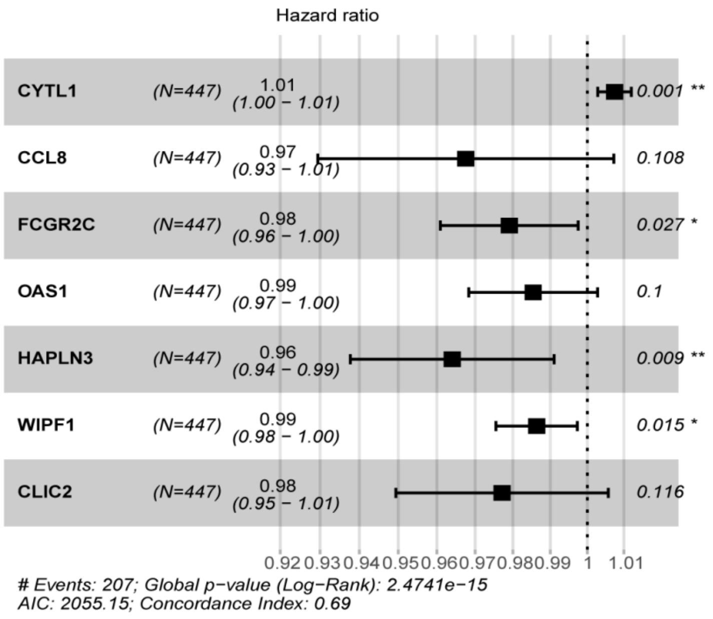

Supplementary Figure 3. The forest plot for the 7-gene prognostic signature.

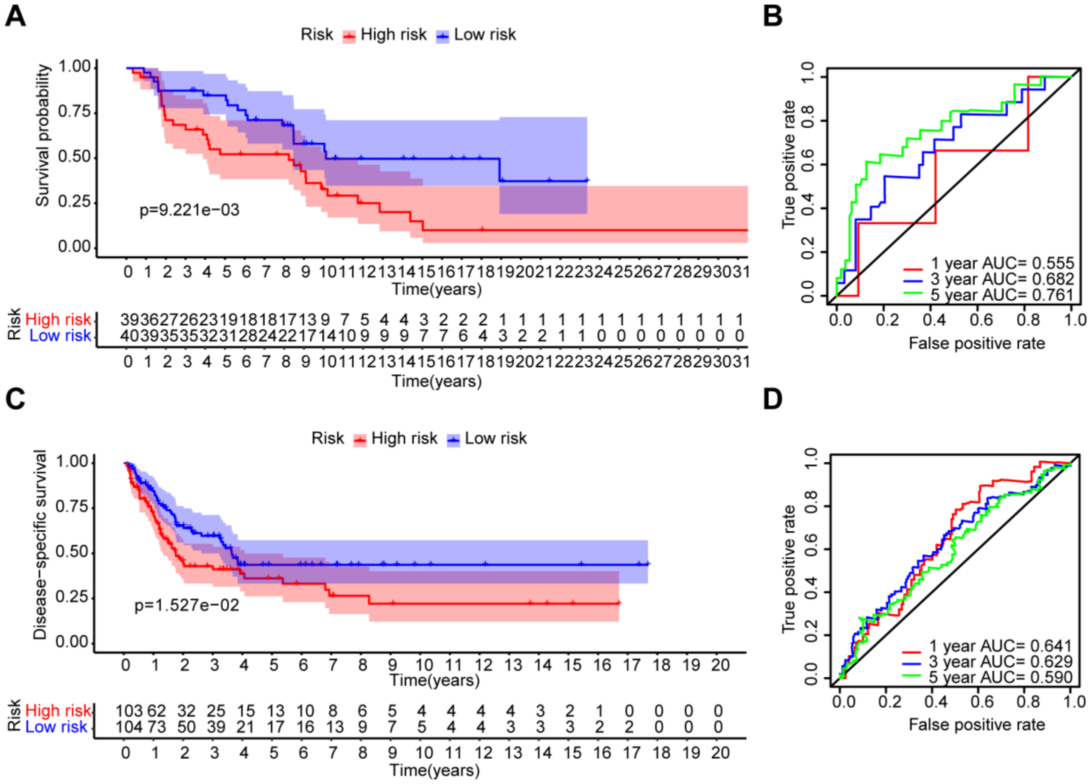

Supplementary Figure 4. Validation of the 7-gene prognostic signature. (**A**, **B**) Kaplan-Meier survival curves and ROC curves of OS in GSE54467. (**C**, **D**) Kaplan-Meier survival curves and ROC curves of DSS in GSE65904.

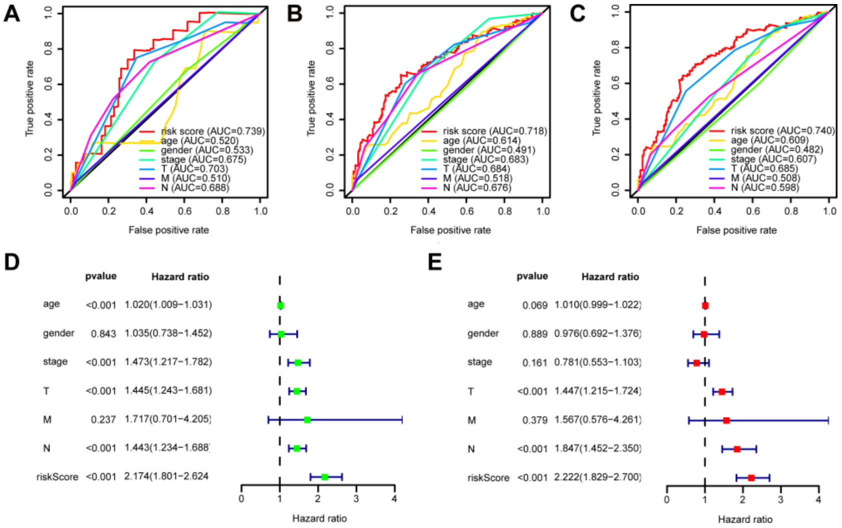

Supplementary Figure 5. Assessment of the independent prognostic value of the prognostic signature. The (**A**) 1-year ROC curves, (**B**) 3-year ROC curves, and (**C**) 5-years ROC curves for the signature and clinical features. (**D**) The univariate Cox regression analysis. (E) The multivariate Cox regression analysis.

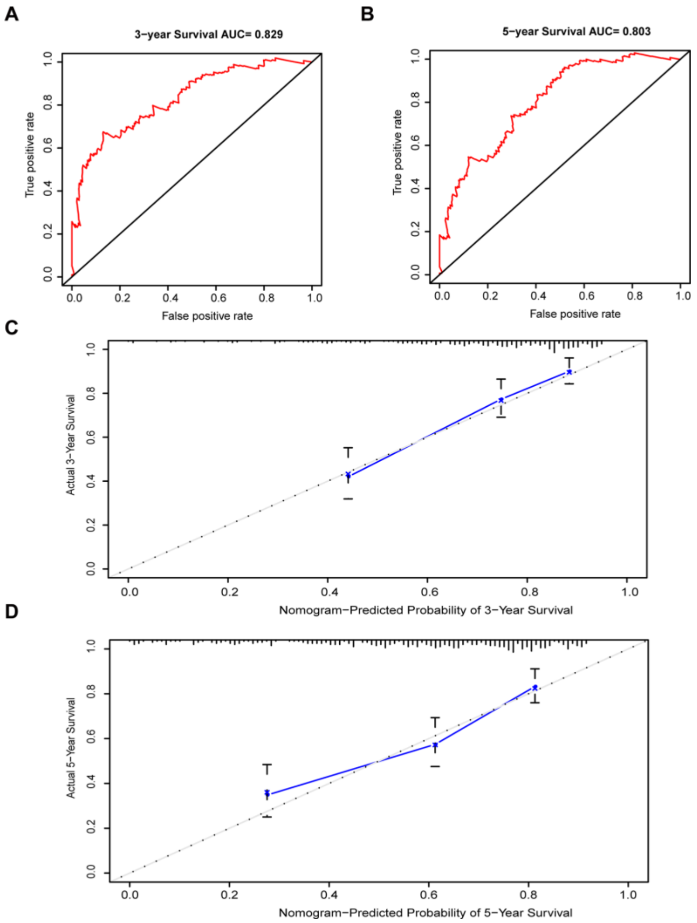

Supplementary Figure 6. Evaluation of the OS nomogram. (**A**, **B**) The (**A**) 3-year and (**B**) 5-year ROC curves. (**C**, **D**) The calibration curves for the probability of (**C**) 3-year and (**D**) 5-year OS.

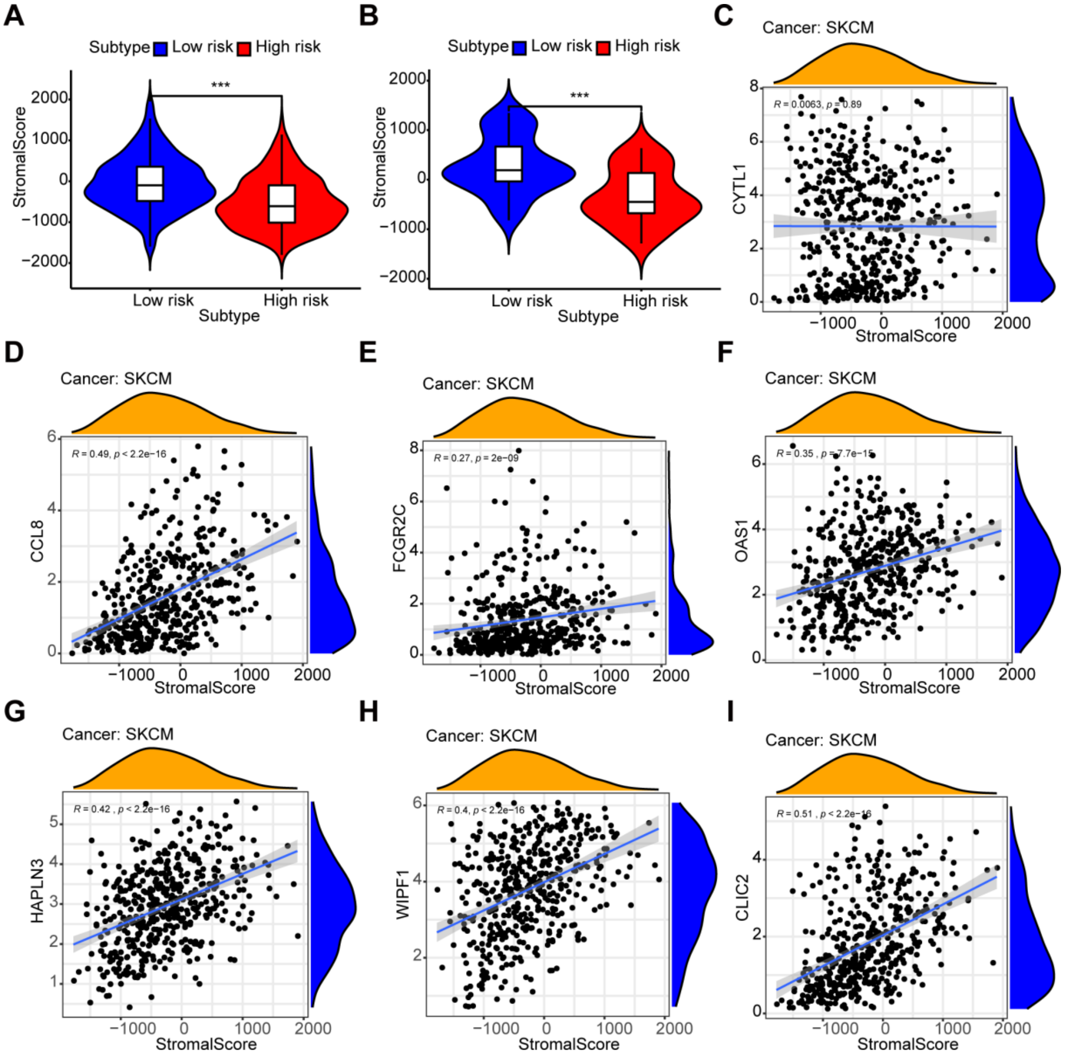

Supplementary Figure 7. Relationship between the prognostic signature and stromal score. (**A**, **B**) The high-risk group has lower stromal scores compared with the low-risk group in (**A**) the TCGA dataset and (**B**) GSE54467. (**C**–**I**) The association between stromal score and the expression of each gene in the 7-gene prognostic signature: (**C**) CYTL1, (**D**) CCL8, (**E**) FCGR2C, (**F**) OAS1, (**G**) HAPLN3, (**H**) WIPF1, (**I**) CLIC2.

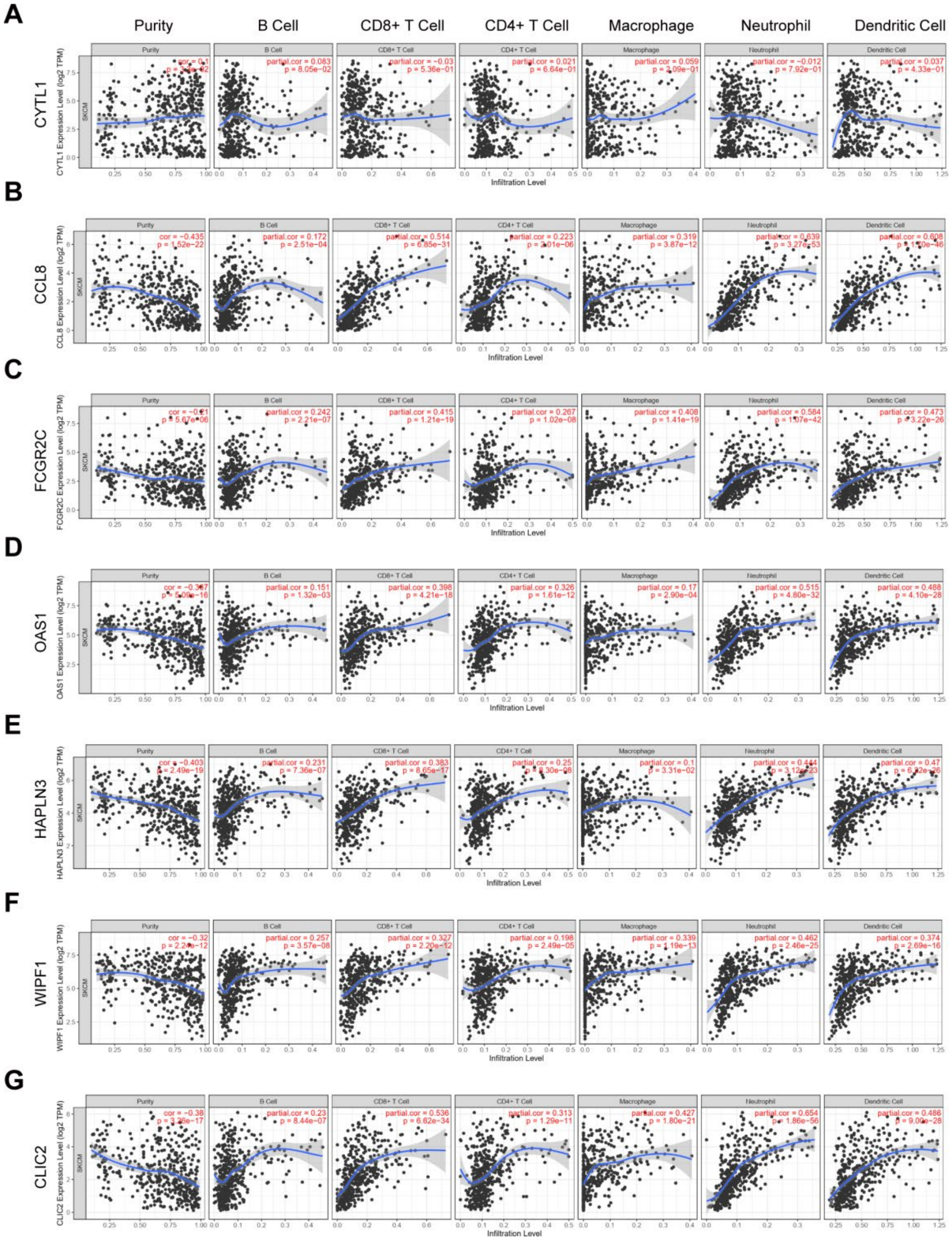

Supplementary Figure 8. Association between the gene expression level and six subtypes of TIICs. (**A**) CYTL1, (**B**) CCL8, (**C**) FCGR2C, (**D**) OAS1, (**E**) HAPLN3, (**F**) WIPF1, (**G**) CLIC2.
