# Supplementary material for: Exploration of the immune cell infiltration-related gene signature in the prognosis of melanoma
Source: Aging (Albany NY). 2021 Jan 10;13(3):3459–82. doi: 10.18632/aging.202279 (PMC7906183; doi:10.18632/aging.202279)
Supplement: Supplementary Table 1 [file aging-13-202279-s002.docx]

| **Supplementary Table 1. Kaplan-Meier survival analysis.** | | | | |
| --- | --- | --- | --- | --- |
| **Number** | **Gene** | **TCGA (p-value)** |  | |
| \| 1 \| \| --- \| \| 2 \| \| 3 \| \| 4 \| \| 5 \| \| 6 \| \| 7 \| \| 8 \| \| 9 \| \| 10 \| \| 11 \| \| 12 \| \| 13 \| \| 14 \| \| 15 \| \| 16 \| \| 17 \| \| 18 \| \| 19 \| \| 20 \| \| 21 \| \| 22 \| \| 23 \| \| 24 \| \| 25 \| \| 26 \| \| 27 \| \| 28 \| \| 29 \| \| 30 \| \| 31 \| \| 32 \| \| 33 \| \| 34 \| \| 35 \| \| 36 \| \| 37 \| \| 38 \| \| 39 \| \| 40 \| \| 41 \| \| 42 \| \| 43 \| \| 44 \| \| 45 \| \| 46 \| \| 47 \| \| 48 \| \| 49 \| \| 50 \| \| 51 \| \| 52 \| \| 53 \| \| 54 \| \| 55 \| \| 56 \| \| 57 \| \| 58 \| \| 59 \| \| 60 \| \| 61 \| \| 62 \| \| 63 \| \| 64 \| \| 65 \| \| 66 \| \| 67 \| \| 68 \| \| 69 \| \| 70 \| \| 71 \| \| 72 \| \| 73 \| \| 74 \| \| 75 \| \| 76 \| \| 77 \| \| 78 \| \| 79 \| \| 80 \| \| 81 \| \| 82 \| \| 83 \| \| 84 \| \| 85 \| \| 86 \| \| 87 \| \| 88 \| \| 89 \| \| 90 \| \| 91 \| \| 92 \| \| 93 \| \| 94 \| \| 95 \| \| 96 \| \| 97 \| \| 98 \| \| 99 \| \| 100 \| \| 101 \| \| 102 \| \| 103 \| \| 104 \| \| 105 \| \| 106 \| \| 107 \| \| 108 \| \| 109 \| \| 110 \| \| 111 \| \| 112 \| \| 113 \| \| 114 \| \| 115 \| \| 116 \| \| 117 \| \| 118 \| \| 119 \| \| 120 \| \| 121 \| \| 122 \| \| 123 \| \| 124 \| \| 125 \| \| 126 \| \| 127 \| \| 128 \| \| 129 \| \| 130 \| \| 131 \| \| 132 \| \| 133 \| \| 134 \| \| 135 \| \| 136 \| \| 137 \| \| 138 \| \| 139 \| \| 140 \| \| 141 \| \| 142 \| \| 143 \| \| 144 \| \| 145 \| \| 146 \| \| 147 \| \| 148 \| \| 149 \| \| 150 \| \| 151 \| \| 152 \| \| 153 \| \| 154 \| \| 155 \| \| 156 \| \| 157 \| \| 158 \| \| 159 \| \| 160 \| \| 161 \| \| 162 \| \| 163 \| \| 164 \| \| 165 \| \| 166 \| \| 167 \| \| 168 \| \| 169 \| \| 170 \| \| 171 \| \| 172 \| \| 173 \| \| 174 \| \| 175 \| \| 176 \| \| 177 \| \| 178 \| \| 179 \| \| 180 \| \| 181 \| \| 182 \| \| 183 \| \| 184 \| \| 185 \| \| 186 \| \| 187 \| \| 188 \| \| 189 \| \| 190 \| \| 191 \| \| 192 \| \| 193 \| \| 194 \| \| 195 \| \| 196 \| \| 197 \| \| 198 \| \| 199 \| \| 200 \| \| 201 \| \| 202 \| \| 203 \| \| 204 \| \| 205 \| \| 206 \| \| 207 \| \| 208 \| \| 209 \| \| 210 \| \| 211 \| \| 212 \| \| 213 \| \| 214 \| \| 215 \| \| 216 \| \| 217 \| \| 218 \| \| 219 \| \| 220 \| \| 221 \| \| 222 \| \| 223 \| \| 224 \| \| 225 \| \| 226 \| \| 227 \| \| 228 \| \| 229 \| \| 230 \| \| 231 \| \| 232 \| \| 233 \| \| 234 \| \| 235 \| \| 236 \| \| 237 \| \| 238 \| \| 239 \| \| 240 \| \| 241 \| \| 242 \| \| 243 \| \| 244 \| \| 245 \| \| 246 \| \| 247 \| \| 248 \| \| 249 \| \| 250 \| \| 251 \| \| 252 \| \| 253 \| \| 254 \| \| 255 \| \| 256 \| \| 257 \| \| 258 \| \| 259 \| \| 260 \| \| 261 \| \| 262 \| \| 263 \| \| 264 \| \| 265 \| \| 266 \| \| 267 \| \| 268 \| \| 269 \| \| 270 \| \| 271 \| \| 272 \| \| 273 \| \| 274 \| \| 275 \| \| 276 \| \| 277 \| \| 278 \| \| 279 \| \| 280 \| \| 281 \| \| 282 \| \| 283 \| \| 284 \| \| 285 \| \| 286 \| \| 287 \| \| 288 \| \| 289 \| \| 290 \| \| 291 \| \| 292 \| \| 293 \| \| 294 \| \| 295 \| \| 296 \| \| 297 \| \| 298 \| \| 299 \| \| 300 \| \| 301 \| \| 302 \| \| 303 \| \| 304 \| \| 305 \| \| 306 \| \| 307 \| \| 308 \| \| 309 \| \| 310 \| \| 311 \| \| 312 \| \| 313 \| \| 314 \| \| 315 \| \| 316 \| \| 317 \| \| 318 \| \| 319 \| \| 320 \| \| 321 \| \| 322 \| \| 323 \| \| 324 \| \| 325 \| \| 326 \| \| 327 \| \| 328 \| \| 329 \| \| 330 \| \| 331 \| \| 332 \| \| 333 \| \| 334 \| \| 335 \| \| 336 \| \| 337 \| \| 338 \| \| 339 \| \| 340 \| \| 341 \| \| 342 \| \| 343 \| \| 344 \| \| 345 \| \| 346 \| \| 347 \| \| 348 \| \| 349 \| \| 350 \| \| 351 \| \| 352 \| \| 353 \| \| 354 \| \| 355 \| \| 356 \| \| 357 \| \| 358 \| \| 359 \| \| 360 \| \| 361 \| \| 362 \| \| 363 \| \| 364 \| \| 365 \| \| 366 \| \| 367 \| \| 368 \| \| 369 \| \| 370 \| \| 371 \| \| 372 \| \| 373 \| \| 374 \| \| 375 \| \| 376 \| \| 377 \| \| 378 \| \| 379 \| \| 380 \| \| 381 \| \| 382 \| \| 383 \| \| 384 \| \| 385 \| \| 386 \| \| 387 \| \| 388 \| \| 389 \| \| 390 \| \| 391 \| \| 392 \| \| 393 \| \| 394 \| \| 395 \| \| 396 \| \| 397 \| \| 398 \| \| 399 \| \| 400 \| \| 401 \| \| 402 \| \| 403 \| \| 404 \| \| 405 \| \| 406 \| \| 407 \| \| 408 \| \| 409 \| \| 410 \| \| 411 \| \| 412 \| \| 413 \| \| 414 \| \| 415 \| \| 416 \| \| 417 \| \| 418 \| \| 419 \| \| 420 \| \| 421 \| \| 422 \| \| 423 \| \| 424 \| \| 425 \| \| 426 \| \| 427 \| \| 428 \| \| 429 \| \| 430 \| \| 431 \| \| 432 \| \| 433 \| \| 434 \| \| 435 \| \| 436 \| \| 437 \| \| 438 \| \| 439 \| \| 440 \| \| 441 \| \| 442 \| \| 443 \| \| 444 \| \| 445 \| \| 446 \| \| 447 \| \| 448 \| \| 449 \| \| 450 \| \| 451 \| \| 452 \| \| 453 \| \| 454 \| \| 455 \| \| 456 \| \| 457 \| \| 458 \| \| 459 \| \| 460 \| \| 461 \| \| 462 \| \| 463 \| \| 464 \| \| 465 \| \| 466 \| \| 467 \| \| 468 \| \| 469 \| \| 470 \| \| 471 \| \| 472 \| \| 473 \| \| 474 \| \| 475 \| \| 476 \| \| 477 \| \| 478 \| \| 479 \| \| 480 \| \| 481 \| \| 482 \| \| 483 \| \| 484 \| \| 485 \| \| 486 \| \| 487 \| \| 488 \| \| 489 \| \| 490 \| \| 491 \| \| 492 \| \| 493 \| \| 494 \| \| 495 \| \| 496 \| \| 497 \| \| 498 \| \| 499 \| \| 500 \| \| 501 \| \| 502 \| \| 503 \| \| 504 \| \| 505 \| \| 506 \| \| 507 \| \| 508 \| \| 509 \| | \| GBP2 \| \| --- \| \| GBP5 \| \| EAF2 \| \| APOBEC3G \| \| SIGLEC10 \| \| STAT1 \| \| FCGR2C \| \| OAS1 \| \| SEMA4D \| \| PARP12 \| \| TRIM22 \| \| IRF1 \| \| IFITM1 \| \| HAPLN3 \| \| SAMD9L \| \| CD86 \| \| PARP9 \| \| APOL6 \| \| CCL8 \| \| BIRC3 \| \| IL21R \| \| TIMD4 \| \| GBP4 \| \| APOL1 \| \| PTPN7 \| \| TLR2 \| \| FCGR2A \| \| FGL2 \| \| RARRES3 \| \| HLA-DPA1 \| \| LAT2 \| \| GCH1 \| \| TNFRSF9 \| \| IL2RA \| \| FCRL3 \| \| SH2D1A \| \| MS4A6A \| \| NCF1 \| \| LAP3 \| \| IFI44L \| \| PARP15 \| \| C3AR1 \| \| CXCL11 \| \| HLA-DOA \| \| ARHGAP9 \| \| SOD2 \| \| HLA-DQA1 \| \| HLA-DOB \| \| CD38 \| \| BST2 \| \| SLC7A7 \| \| ARID5A \| \| GIMAP7 \| \| PTPN22 \| \| TNFSF10 \| \| NR1H3 \| \| LST1 \| \| SRGN \| \| CD274 \| \| P2RY10 \| \| CLEC7A \| \| HAVCR2 \| \| MNDA \| \| HLA-DRA \| \| HLA-DMB \| \| PTPRC \| \| TLR8 \| \| PLA2G2D \| \| PARP14 \| \| TRAF3IP3 \| \| SP140 \| \| IL2RG \| \| CCR1 \| \| SIT1 \| \| PDCD1LG2 \| \| HLA-DPB1 \| \| IRF2 \| \| HLA-DRB1 \| \| NCF1C \| \| IL15RA \| \| IFNG \| \| EVI2B \| \| IL4I1 \| \| PTPN6 \| \| GBP1 \| \| NMI \| \| CSF1 \| \| ALOX5 \| \| TLR10 \| \| HLA-DQB1 \| \| CD74 \| \| GZMB \| \| CXCL10 \| \| FASLG \| \| NKG7 \| \| CTSS \| \| SPOCK2 \| \| ARHGAP30 \| \| SIRPG \| \| APOL2 \| \| CSF1R \| \| APOL3 \| \| ARHGAP25 \| \| HLA-B \| \| UBE2L6 \| \| SLAMF8 \| \| LILRB2 \| \| LCP2 \| \| VNN2 \| \| TMSB4X \| \| CD72 \| \| MYO1F \| \| VAV1 \| \| TMEM176B \| \| HCLS1 \| \| LILRB1 \| \| CD8A \| \| TNFSF13B \| \| PARVG \| \| SERPINA1 \| \| CARD11 \| \| SLA \| \| CD8B \| \| B2M \| \| SPN \| \| CD7 \| \| LAPTM5 \| \| LILRB4 \| \| ALOX5AP \| \| OASL \| \| NCF1B \| \| LAIR1 \| \| RCSD1 \| \| AIF1 \| \| IFIT3 \| \| DOCK2 \| \| LAX1 \| \| IKZF3 \| \| LAG3 \| \| CXCL9 \| \| IFIH1 \| \| CD53 \| \| TNFRSF17 \| \| PSMB9 \| \| CCR2 \| \| SPI1 \| \| WAS \| \| GZMA \| \| INPP5D \| \| CIITA \| \| APBB1IP \| \| RSAD2 \| \| TAPBP \| \| IL18 \| \| CXCR6 \| \| PILRA \| \| SLA2 \| \| TAGAP \| \| LTA \| \| LYZ \| \| BTN3A1 \| \| CD5 \| \| RASGRP1 \| \| PYHIN1 \| \| PRF1 \| \| CD3D \| \| TRIM69 \| \| CD69 \| \| DHRS3 \| \| CXCR3 \| \| SLAMF6 \| \| CLEC4A \| \| CD247 \| \| CCR5 \| \| IL18BP \| \| HSH2D \| \| RASSF4 \| \| WIPF1 \| \| CD84 \| \| LRMP \| \| AOAH \| \| CD3G \| \| AIM2 \| \| POU2AF1 \| \| PIM2 \| \| GIMAP2 \| \| CD28 \| \| GZMK \| \| GIMAP1 \| \| FCGR1A \| \| CYBB \| \| OAS2 \| \| HSD11B1 \| \| POU2F2 \| \| RNASE6 \| \| GMFG \| \| BTN3A3 \| \| GNLY \| \| VAMP5 \| \| GIMAP4 \| \| TYROBP \| \| CD3E \| \| IL2RB \| \| ITGAL \| \| PSTPIP1 \| \| CXCL13 \| \| IRF8 \| \| IL7R \| \| BANK1 \| \| ITGAM \| \| TRAT1 \| \| MSR1 \| \| CD79A \| \| VCAM1 \| \| CD2 \| \| CD300LF \| \| EPSTI1 \| \| ANKRD22 \| \| C1QB \| \| ITGB2 \| \| HLA-C \| \| C1QA \| \| LCK \| \| FBP1 \| \| CD300A \| \| FGD2 \| \| PIK3AP1 \| \| IL32 \| \| RELB \| \| GIMAP6 \| \| HLA-DMA \| \| GZMH \| \| NAPSB \| \| SAMSN1 \| \| TRIM21 \| \| TBC1D10C \| \| DTX3L \| \| UBASH3A \| \| LCP1 \| \| WARS \| \| SKAP1 \| \| ADAMDEC1 \| \| TAPBPL \| \| CCL5 \| \| PTK2B \| \| ITM2A \| \| PTGER4 \| \| C1QC \| \| IRF7 \| \| HCK \| \| FBXO6 \| \| CD48 \| \| CD96 \| \| LTB \| \| SELPLG \| \| OSCAR \| \| CD163 \| \| CD14 \| \| DENND1C \| \| CCND2 \| \| CST7 \| \| TNFRSF18 \| \| HLA-DQB2 \| \| HK3 \| \| HLA-DRB5 \| \| FPR1 \| \| PLEK \| \| CD40 \| \| CTLA4 \| \| PSME2 \| \| HCP5 \| \| IGSF6 \| \| PTAFR \| \| NCF4 \| \| RASSF5 \| \| ZBED2 \| \| CD6 \| \| SELL \| \| PDCD1 \| \| DOK2 \| \| C2 \| \| CORO1A \| \| TRAFD1 \| \| IL10RA \| \| RAB37 \| \| CD4 \| \| IFI27 \| \| HLA-E \| \| TNFAIP8L2 \| \| NCF2 \| \| KLRB1 \| \| RAC2 \| \| ADAM8 \| \| MS4A1 \| \| SLAMF1 \| \| JSRP1 \| \| PSME1 \| \| ABI3 \| \| CCL2 \| \| SERPING1 \| \| DOCK8 \| \| ICAM1 \| \| BATF2 \| \| P2RY13 \| \| SLAMF7 \| \| HLA-F \| \| MFNG \| \| ICAM3 \| \| MS4A4A \| \| CR2 \| \| VSIG4 \| \| GBP3 \| \| P2RY8 \| \| IL12RB1 \| \| MPEG1 \| \| ICOS \| \| SLC15A3 \| \| APOL4 \| \| EVI2A \| \| CLEC10A \| \| HLA-DRB6 \| \| LRRC25 \| \| FGR \| \| SYTL3 \| \| LGALS9 \| \| FCER1G \| \| SUSD3 \| \| BTK \| \| GIMAP8 \| \| CD37 \| \| PSD4 \| \| PSMB10 \| \| CLIC2 \| \| TIMP1 \| \| TNFRSF4 \| \| ITK \| \| BTN3A2 \| \| PARP10 \| \| UBD \| \| HLA-A \| \| CYTL1 \| \| TNFRSF1B \| \| ACSL5 \| \| HCST \| \| LY86 \| \| EBI3 \| \| ABCG1 \| \| UNC93B1 \| \| NCR3 \| \| STK17B \| \| IFITM3 \| \| CD40LG \| \| RARRES1 \| \| TAP1 \| \| RRAS \| \| BCL3 \| \| CD19 \| \| MYO1G \| \| ARRB2 \| \| SEMA6A \| \| CTSW \| \| RBP5 \| \| ZAP70 \| \| MVP \| \| NFKBIA \| \| CA6 \| \| LAMP3 \| \| TNFAIP8 \| \| FLI1 \| \| SLCO2B1 \| \| MARCO \| \| RNASET2 \| \| IFI35 \| \| FMNL1 \| \| ISG15 \| \| FCN1 \| \| FCER2 \| \| ETV7 \| \| GPR18 \| \| PRDM1 \| \| SIGLEC8 \| \| PLEKHO1 \| \| MAN1A1 \| \| CD209 \| \| LGMN \| \| VPREB3 \| \| CCDC69 \| \| TOX \| \| RASGRP2 \| \| PLCB2 \| \| CD52 \| \| DGKA \| \| ARHGDIB \| \| CMKLR1 \| \| CTSC \| \| CYFIP2 \| \| SLC40A1 \| \| TNFAIP2 \| \| SOCS1 \| \| CCR7 \| \| FAM107B \| \| CST3 \| \| LPXN \| \| CPNE5 \| \| UCP2 \| \| IL33 \| \| TCL1A \| \| BCAN \| \| G0S2 \| \| ARL4C \| \| CXCL16 \| \| PSMB8 \| \| KLHL6 \| \| CDC42SE2 \| \| BLK \| \| IFI44 \| \| C3 \| \| LSP1 \| \| ITGAX \| \| OPTN \| \| GATA3 \| \| LYN \| \| FGD3 \| \| MAFB \| \| CCL3 \| \| CD5L \| \| DERL3 \| \| DENND2D \| \| ATP2A3 \| \| SLC29A3 \| \| CTGF \| \| HLA-H \| \| GNA15 \| \| GALM \| \| TMC8 \| \| ARHGAP4 \| \| JUNB \| \| BTG2 \| \| SFTPC \| \| SH2D2A \| \| ALDH2 \| \| MS4A7 \| \| SIPA1 \| \| SEMA4A \| \| PLAC8 \| \| MRC1 \| \| CA14 \| \| STX11 \| \| UNC13D \| \| SYK \| \| GAB2 \| \| TAP2 \| \| CD83 \| \| TSPAN33 \| \| RGS16 \| \| AKNA \| \| SPINT2 \| \| CD1C \| \| FOLR2 \| \| RARRES2 \| \| C1S \| \| CNTFR \| \| CNN2 \| \| EPHB3 \| \| SYTL1 \| \| RPS6KA1 \| \| GPR132 \| \| ALOX15B \| \| TMSB10 \| \| IL3RA \| \| LGALS2 \| \| LIPA \| \| TREM2 \| \| APOC1 \| \| TGM2 \| \| DYSF \| \| ITGB7 \| \| SPIB \| \| SH2D3C \| \| IL1B \| \| GADD45B \| \| MX1 \| \| HS3ST2 \| \| PIM1 \| \| C7 \| \| WNK4 \| \| TCIRG1 \| \| THBS1 \| \| C1R \| \| FOXF2 \| \| LBH \| \| CYP1B1 \| \| IRF5 \| \| CEBPA \| \| IFI6 \| \| TMEM37 \| \| DEF6 \| \| ERBB3 \| \| EDNRB \| \| VMO1 \| \| BZW2 \| \| CASP1 \| \| BCHE \| \| MAP4K1 \| \| OAS3 \| \| GZMM \| \| CXCL12 \| \| RHOG \| \| C5AR1 \| | \| 1.07E-08 \| \| --- \| \| 2.62E-08 \| \| 2.88E-08 \| \| 3.49E-08 \| \| 4.40E-08 \| \| 4.83E-08 \| \| 6.25E-08 \| \| 8.89E-08 \| \| 1.04E-07 \| \| 1.18E-07 \| \| 1.21E-07 \| \| 1.35E-07 \| \| 1.47E-07 \| \| 1.49E-07 \| \| 1.51E-07 \| \| 1.80E-07 \| \| 2.06E-07 \| \| 2.08E-07 \| \| 2.23E-07 \| \| 2.30E-07 \| \| 2.70E-07 \| \| 3.02E-07 \| \| 3.07E-07 \| \| 3.87E-07 \| \| 3.90E-07 \| \| 3.91E-07 \| \| 3.94E-07 \| \| 4.85E-07 \| \| 5.42E-07 \| \| 5.72E-07 \| \| 5.77E-07 \| \| 6.40E-07 \| \| 6.43E-07 \| \| 7.09E-07 \| \| 7.37E-07 \| \| 8.55E-07 \| \| 9.67E-07 \| \| 9.85E-07 \| \| 9.89E-07 \| \| 1.11E-06 \| \| 1.14E-06 \| \| 1.15E-06 \| \| 1.25E-06 \| \| 1.26E-06 \| \| 1.34E-06 \| \| 1.36E-06 \| \| 1.40E-06 \| \| 1.47E-06 \| \| 1.51E-06 \| \| 1.52E-06 \| \| 1.55E-06 \| \| 1.61E-06 \| \| 1.63E-06 \| \| 1.65E-06 \| \| 1.87E-06 \| \| 1.87E-06 \| \| 1.93E-06 \| \| 2.03E-06 \| \| 2.18E-06 \| \| 2.23E-06 \| \| 2.30E-06 \| \| 2.34E-06 \| \| 2.37E-06 \| \| 2.43E-06 \| \| 2.45E-06 \| \| 2.49E-06 \| \| 2.51E-06 \| \| 2.53E-06 \| \| 2.58E-06 \| \| 2.58E-06 \| \| 2.85E-06 \| \| 2.89E-06 \| \| 3.05E-06 \| \| 3.12E-06 \| \| 3.19E-06 \| \| 3.27E-06 \| \| 3.72E-06 \| \| 3.89E-06 \| \| 3.91E-06 \| \| 4.13E-06 \| \| 4.19E-06 \| \| 4.21E-06 \| \| 4.28E-06 \| \| 4.40E-06 \| \| 4.49E-06 \| \| 4.64E-06 \| \| 4.82E-06 \| \| 5.06E-06 \| \| 5.20E-06 \| \| 5.21E-06 \| \| 5.23E-06 \| \| 5.62E-06 \| \| 5.82E-06 \| \| 5.93E-06 \| \| 6.08E-06 \| \| 6.39E-06 \| \| 6.55E-06 \| \| 6.65E-06 \| \| 6.67E-06 \| \| 6.71E-06 \| \| 6.74E-06 \| \| 6.78E-06 \| \| 6.82E-06 \| \| 7.20E-06 \| \| 7.20E-06 \| \| 7.27E-06 \| \| 7.46E-06 \| \| 7.75E-06 \| \| 7.84E-06 \| \| 7.88E-06 \| \| 7.95E-06 \| \| 8.15E-06 \| \| 8.44E-06 \| \| 8.64E-06 \| \| 8.75E-06 \| \| 8.90E-06 \| \| 9.04E-06 \| \| 9.18E-06 \| \| 9.25E-06 \| \| 9.43E-06 \| \| 9.88E-06 \| \| 1.08E-05 \| \| 1.09E-05 \| \| 1.09E-05 \| \| 1.11E-05 \| \| 1.15E-05 \| \| 1.16E-05 \| \| 1.18E-05 \| \| 1.21E-05 \| \| 1.26E-05 \| \| 1.29E-05 \| \| 1.30E-05 \| \| 1.31E-05 \| \| 1.34E-05 \| \| 1.39E-05 \| \| 1.56E-05 \| \| 1.56E-05 \| \| 1.59E-05 \| \| 1.59E-05 \| \| 1.60E-05 \| \| 1.60E-05 \| \| 1.64E-05 \| \| 1.65E-05 \| \| 1.68E-05 \| \| 1.69E-05 \| \| 1.70E-05 \| \| 1.76E-05 \| \| 1.76E-05 \| \| 1.79E-05 \| \| 1.86E-05 \| \| 1.88E-05 \| \| 1.93E-05 \| \| 1.93E-05 \| \| 1.96E-05 \| \| 2.02E-05 \| \| 2.03E-05 \| \| 2.06E-05 \| \| 2.09E-05 \| \| 2.12E-05 \| \| 2.14E-05 \| \| 2.17E-05 \| \| 2.21E-05 \| \| 2.25E-05 \| \| 2.28E-05 \| \| 2.30E-05 \| \| 2.33E-05 \| \| 2.36E-05 \| \| 2.40E-05 \| \| 2.44E-05 \| \| 2.45E-05 \| \| 2.46E-05 \| \| 2.47E-05 \| \| 2.47E-05 \| \| 2.63E-05 \| \| 2.77E-05 \| \| 2.78E-05 \| \| 2.83E-05 \| \| 2.86E-05 \| \| 2.87E-05 \| \| 2.91E-05 \| \| 2.94E-05 \| \| 3.03E-05 \| \| 3.03E-05 \| \| 3.08E-05 \| \| 3.15E-05 \| \| 3.23E-05 \| \| 3.26E-05 \| \| 3.30E-05 \| \| 3.32E-05 \| \| 3.36E-05 \| \| 3.45E-05 \| \| 3.49E-05 \| \| 3.52E-05 \| \| 3.67E-05 \| \| 3.82E-05 \| \| 3.84E-05 \| \| 3.85E-05 \| \| 3.88E-05 \| \| 3.96E-05 \| \| 4.09E-05 \| \| 4.10E-05 \| \| 4.19E-05 \| \| 4.35E-05 \| \| 4.40E-05 \| \| 4.50E-05 \| \| 4.58E-05 \| \| 4.68E-05 \| \| 4.71E-05 \| \| 4.81E-05 \| \| 4.90E-05 \| \| 4.93E-05 \| \| 5.27E-05 \| \| 5.28E-05 \| \| 5.47E-05 \| \| 5.50E-05 \| \| 5.65E-05 \| \| 5.66E-05 \| \| 5.70E-05 \| \| 6.03E-05 \| \| 6.11E-05 \| \| 6.40E-05 \| \| 6.41E-05 \| \| 6.56E-05 \| \| 6.66E-05 \| \| 6.77E-05 \| \| 6.82E-05 \| \| 6.83E-05 \| \| 7.10E-05 \| \| 7.30E-05 \| \| 7.30E-05 \| \| 7.36E-05 \| \| 7.40E-05 \| \| 7.48E-05 \| \| 7.50E-05 \| \| 7.51E-05 \| \| 7.58E-05 \| \| 7.85E-05 \| \| 8.18E-05 \| \| 8.27E-05 \| \| 8.28E-05 \| \| 8.41E-05 \| \| 8.47E-05 \| \| 8.50E-05 \| \| 8.81E-05 \| \| 9.08E-05 \| \| 9.30E-05 \| \| 9.63E-05 \| \| 9.65E-05 \| \| 9.89E-05 \| \| 1.00E-04 \| \| 0.0001 \| \| 0.0001 \| \| 0.000103 \| \| 0.000106 \| \| 0.000106 \| \| 0.00011 \| \| 0.00011 \| \| 0.000111 \| \| 0.000112 \| \| 0.000119 \| \| 0.00012 \| \| 0.000121 \| \| 0.000123 \| \| 0.000125 \| \| 0.000127 \| \| 0.000127 \| \| 0.000128 \| \| 0.000129 \| \| 0.000129 \| \| 0.000129 \| \| 0.000132 \| \| 0.000137 \| \| 0.000143 \| \| 0.000143 \| \| 0.000146 \| \| 0.000147 \| \| 0.000148 \| \| 0.000149 \| \| 0.000152 \| \| 0.00016 \| \| 0.000163 \| \| 0.000167 \| \| 0.00017 \| \| 0.000178 \| \| 0.000178 \| \| 0.000178 \| \| 0.000178 \| \| 0.000179 \| \| 0.000179 \| \| 0.000183 \| \| 0.000184 \| \| 0.000196 \| \| 0.000204 \| \| 0.00021 \| \| 0.000215 \| \| 0.000218 \| \| 0.000219 \| \| 0.000219 \| \| 0.000229 \| \| 0.000231 \| \| 0.00024 \| \| 0.000243 \| \| 0.000246 \| \| 0.000254 \| \| 0.00027 \| \| 0.000272 \| \| 0.000272 \| \| 0.000274 \| \| 0.000295 \| \| 0.000296 \| \| 0.000298 \| \| 0.0003 \| \| 0.000301 \| \| 0.000303 \| \| 0.000306 \| \| 0.000309 \| \| 0.000318 \| \| 0.000334 \| \| 0.000336 \| \| 0.000347 \| \| 0.000362 \| \| 0.000366 \| \| 0.000375 \| \| 0.000415 \| \| 0.00042 \| \| 0.000422 \| \| 0.000429 \| \| 0.000455 \| \| 0.000471 \| \| 0.000473 \| \| 0.000492 \| \| 0.000494 \| \| 0.000495 \| \| 0.000508 \| \| 0.000539 \| \| 0.000545 \| \| 0.00055 \| \| 0.000573 \| \| 0.000574 \| \| 0.000609 \| \| 0.000653 \| \| 0.00067 \| \| 0.000677 \| \| 0.000696 \| \| 0.000703 \| \| 0.000748 \| \| 0.000803 \| \| 0.000812 \| \| 0.00083 \| \| 0.000832 \| \| 0.000845 \| \| 0.000938 \| \| 0.000942 \| \| 0.00098 \| \| 0.001047 \| \| 0.001059 \| \| 0.001069 \| \| 0.001072 \| \| 0.001193 \| \| 0.001209 \| \| 0.001267 \| \| 0.001286 \| \| 0.001372 \| \| 0.001372 \| \| 0.001499 \| \| 0.001614 \| \| 0.001623 \| \| 0.001626 \| \| 0.001673 \| \| 0.001685 \| \| 0.001778 \| \| 0.001793 \| \| 0.001816 \| \| 0.001884 \| \| 0.001917 \| \| 0.001941 \| \| 0.001957 \| \| 0.002081 \| \| 0.00211 \| \| 0.002144 \| \| 0.002155 \| \| 0.002214 \| \| 0.002471 \| \| 0.002562 \| \| 0.002577 \| \| 0.002631 \| \| 0.002678 \| \| 0.002717 \| \| 0.002823 \| \| 0.002924 \| \| 0.003145 \| \| 0.003223 \| \| 0.003266 \| \| 0.003479 \| \| 0.003705 \| \| 0.003718 \| \| 0.003869 \| \| 0.003954 \| \| 0.003969 \| \| 0.004016 \| \| 0.004248 \| \| 0.004291 \| \| 0.004364 \| \| 0.004423 \| \| 0.004467 \| \| 0.004751 \| \| 0.004811 \| \| 0.004994 \| \| 0.005064 \| \| 0.00507 \| \| 0.00508 \| \| 0.005094 \| \| 0.005161 \| \| 0.005254 \| \| 0.005376 \| \| 0.005385 \| \| 0.005427 \| \| 0.005521 \| \| 0.005534 \| \| 0.005801 \| \| 0.005816 \| \| 0.006031 \| \| 0.006109 \| \| 0.006152 \| \| 0.006537 \| \| 0.006573 \| \| 0.006779 \| \| 0.007071 \| \| 0.007366 \| \| 0.007419 \| \| 0.007707 \| \| 0.007746 \| \| 0.008256 \| \| 0.009061 \| \| 0.009203 \| \| 0.009342 \| \| 0.010128 \| \| 0.010362 \| \| 0.010529 \| \| 0.010722 \| \| 0.010809 \| \| 0.011492 \| \| 0.011848 \| \| 0.01242 \| \| 0.012529 \| \| 0.012587 \| \| 0.012886 \| \| 0.012898 \| \| 0.012923 \| \| 0.013073 \| \| 0.014164 \| \| 0.014639 \| \| 0.014705 \| \| 0.015069 \| \| 0.015097 \| \| 0.016269 \| \| 0.016498 \| \| 0.016841 \| \| 0.017028 \| \| 0.018113 \| \| 0.018411 \| \| 0.018582 \| \| 0.020083 \| \| 0.021352 \| \| 0.02149 \| \| 0.021576 \| \| 0.02252 \| \| 0.022971 \| \| 0.023652 \| \| 0.024033 \| \| 0.02524 \| \| 0.02549 \| \| 0.027343 \| \| 0.027452 \| \| 0.028512 \| \| 0.028634 \| \| 0.028979 \| \| 0.02913 \| \| 0.029397 \| \| 0.029492 \| \| 0.029925 \| \| 0.030239 \| \| 0.030603 \| \| 0.030919 \| \| 0.031281 \| \| 0.031604 \| \| 0.031822 \| \| 0.032064 \| \| 0.033065 \| \| 0.033825 \| \| 0.033864 \| \| 0.034923 \| \| 0.035161 \| \| 0.035344 \| \| 0.036498 \| \| 0.036621 \| \| 0.038004 \| \| 0.039826 \| \| 0.040198 \| \| 0.040382 \| \| 0.042208 \| \| 0.042534 \| \| 0.042838 \| \| 0.043514 \| \| 0.043829 \| \| 0.044201 \| \| 0.044838 \| \| 0.045 \| \| 0.047652 \| | |  |
