# Supplementary material for: Exploration of the immune cell infiltration-related gene signature in the prognosis of melanoma
Source: Aging (Albany NY). 2021 Jan 10;13(3):3459–82. doi: 10.18632/aging.202279 (PMC7906183; doi:10.18632/aging.202279)
Supplement: Supplementary Table 2 [file aging-13-202279-s003.docx]

| **Supplementary Table 2. The genes in the** **selected module identified by WGCNA.** | | | | |
| --- | --- | --- | --- | --- |
| **Merged**  **Colors** | | **Genes**  **No.** | | **Names** |
| Blue | 841 | |  | MARCO, DTX3L, CD72, PIK3CD, AKNA, CIITA, CXCL11, TCFL5, NEDD4L, IFI44L, DDR1, BIRC3, VEPH1, MFNG, CCL3, APOL3, UBD, ZYX, TES, BTG1, METTL7B, PTPN6, PTPN18, RRAS, UNC93B1, SLC4A2, SEMA6A, HCLS1, MAD2L1BP, GPSM1, DEF6, ID2, CSNK1E, CD7, CYTL1, HLA-F, MSR1, BYSL, TAPBPL, RELB, BASP1, FOLR2, SLCO2B1, SMOX, SLC2A3, IL10RA, OAS1, PSMB10, TSC22D3, GNLY, HILS1, PRF1, CXCL13, HRK, TNFRSF4, EFHD2, TBC1D16, TSPAN9, ZHX2, RASSF4, MFAP2, KCTD17, TTYH1, CLIC1, NNMT, MAOB, CASP4, TBXA2R, TTLL4, BCL3, TRIB3, LAG3, HCN2, LZTS2, CCL8, KCNE4, TNFSF10, EPAS1, ATF3, GPR161, PVR, SLC37A2, CEBPA, RGS10, RTKN, ENPP2, SAMD9, CCL20, HHEX, CD22, UCP2, ADAMDEC1, EN2, PARVA, TRIM22, FGF1, RGS1, FOSB, TRIM69, SEC14L1, TFPI2, PITRM1, GRB10, TNFRSF1B, NR2F1, SLC4A3, FUCA1, MNDA, PTPRC, LY96, PLA2G2D, KLF15, CYP1B1, IQGAP2, ATN1, PIM2, SLC9A3R1, NDUFS2, TREM2, BIN1, DHRS3, CTSH, IGSF11, NMB, C1RL, SCRIB, FDFT1, EMILIN2, CBX2, GIMAP4, PLEKHG4, TOR3A, PARP10, IRS2, CLEC10A, CCND3, IL1R1, NOMO1, CCL13, RASSF5, EGR2, GBP1, RAB20, RARRES1, CLEC2B, HPS4, SLAMF1, DDB1, HERPUD1, CHI3L2, SLC39A1, SLAMF7, GIMAP2, LIF, LASP1, NFKBIA, GM2A, GAS6, G0S2, EPSTI1, APOL6, ITGAX, ETNK2, PARP9, DUSP1, ARRB2, MS4A4A, VAV3, NFKB2, PLEKHA5, LDLRAD3, ARHGAP4, PFKM, YWHAH, PDGFB, CLSTN3, ZFP36, NKX2-5, MX2, FKBP4, MEGF8, OLFML3, DERL3, STAT5A, SLC1A3, SPI1, OSTF1, DIRAS1, MAFB, VARS, LGALS9, CFH, LCP1, STXBP2, CRTAP, SULF1, IGF1R, USP18, PDCD1, SIPA1, CXCR6, CORO1A, AP1B1, PTPN7, SLFN11, SH2D1A, STAB1, CUL7, IL32, COL22A1, CR2, GIT1, CMKLR1, RHOG, SKAP1, CD300A, HDAC1, ST5, NUP210, CTSC, KLF2, FAM107B, KIAA0040, STK17B, PIK3AP1, TNIP1, CAPZB, NAV2, MS4A1, EVI2B, JUN, RNF44, CXCL16, CTXN1, ABI3, TCN2, ALOX5AP, PILRA, TRIM21, TBX2, CCDC8, PTAFR, NAPSB, CD79B, GADD45G, FCGR2A, VCP, PCGF2, IL12RB1, CNTFR, SERPINB9, PLXND1, CD40, RAC2, RBM38, GOT2, IL24, RTN2, CELSR2, KCNJ10, TYRO3, FBP1, GSTM1, TLR4, PDE3A, CD200, CSK, SEMA7A, P2RY8, MYH10, SLC35B2, TBXAS1, HAPLN3, SLC7A2, CD48, IFI44, SYK, ITGB2, PTGFRN, TCF7, CHKA, DHCR7, IL33, SIRPG, CD3E, LCK, MYD88, GCH1, SLC25A13, FGL2, HLA-DQB2, ARPC3, VPREB3, OGFR, CLEC1A, ERGIC3, ADCY6, DOCK10, CYFIP2, PREX1, OCIAD2, IL3RA, TBC1D10C, GADD45B, ARHGEF6, RNASE6, NOL3, RBCK1, SLC38A1, RGS2, PAX3, RGS16, FCGR2C, ADAM8, IFIT5, PLSCR1, SAMD9L, CD86, B4GALT1, FGD1, PLAUR, CIB1, IRF1, NXPH4, SLC25A23, CCND2, KCTD15, ZMIZ2, IFNGR2, MRPS25, PARD6G, CD79A, PTK7, KLHL21, ARHGAP9, CDC42SE2, B4GALT2, DENND1C, GMIP, NR1H3, CLDN11, PACS1, GZMA, HLA-DQA1, ARL4C, LYN, CSF1, DNASE2, HIST1H4I, HCST, DPYD, POU2AF1, NT5DC2, MS4A6A, SERPINA1, CARD11, CD2, MGST2, ARID5A, GZMB, CCR7, RSU1, C7, IFI35, FBXO6, GIMAP6, SLC15A3, TLR2, INPP5D, GPSM3, GBP4, CMTM3, POLD2, ARPC1A, TNFRSF17, MX1, XKR8, HEG1, METTL7A, C2, ITGA4, GZMH, SLC16A3, ADRA2C, LRP4, ALG8, BTN3A3, TNFRSF13C, ARHGAP30, PITPNM1, NPPC, RAB33A, TAP2, IRF8, CAD, LRRC25, DDX58, CPVL, ADAM19, RNF43, KLF13, PTK2B, PACSIN3, UBC, IFIT1, SLC25A4, CALR, ITM2A, HIST3H2A, LILRB4, CHRNA1, ALDH2, MAN1A1, CD83, THBS1, PLA2G5, CA14, RGL1, PIM1, SLC25A3, MKNK2, ATP6V0E1, FCER2, LSP1, YIPF3, FAM89A, AHCY, SOX12, KRT8, SOCS1, SLAMF6, MS4A7, VAV1, LITAF, APOBEC3G, HCK, BTD, SOD2, EI24, SLC2A6, ISG20, CYBA, HOXC10, ACTR1B, FKBP11, FAM20A, SYDE1, LTB, TRAF1, ITGAL, BTN3A1, CD82, CEBPD, SPTAN1, FRZB, CLU, GBP2, AKR1B1, FCGRT, WWC3, MGAT4B, SLAMF9, CD37, HSD11B1, PPP1R16B, PPM1M, DOK2, LFNG, IL18BP, PYCR1, GREB1, CHMP1B, FYN, APBB1IP, USF1, SCARF2, SNN, IRF2, ZAP70, SERPINB1, CERKL, GBP3, PECAM1, SPTBN2, APBA2, DUSP2, IRX3, TLN1, SEMA6B, SLAMF8, BMF, SREBF2, PPM1H, PTTG1IP, CCDC69, PQLC3, C5AR1, MXRA7, TBC1D7, PSME3, CST7, TMEM97, NME1, PER1, EFNA1, AIM2, NFATC2, HK3, ETV7, SPOCK2, FKBP5, FNBP1, TRAF4, PSMB9, CS, SLC7A1, SNAI2, IL17D, CYBB, FMNL1, RGS19, ACP2, FES, HLA-DOB, CCR5, LGALS2, DOK3, CFD, RSAD2, EHD1, GALNT3, ABCG1, HCP5, MAG, TMEM173, TAGAP, CXCR3, IL4R, CA11, GLDC, SLC44A2, PSME2, TBC1D9, PPIA, RAMP3, ARHGAP25, MYBBP1A, DUSP5, IL27RA, HLA-DRB6, GAB2, LGMN, RASSF2, TNFAIP3, IL1B, LBH, WAS, MTHFD1L, ANKRD22, ARHGAP15, IL4I1, FAM78A, RAC3, DVL3, EPHB4, RFX5, TGM2, APOL2, PPP1R9B, FAS, MMP12, SH3BP4, SHMT2, MGAT1, SCFD2, CGREF1, LRP2, WDR63, PDZRN3, BTN3A2, CPM, PARP14, VSIG4, RAI1, TMC8, JAK1, TNFAIP8L2, ALOX5, PSME1, SLC24A5, CD52, PHB, SLC40A1, EOMES, SLC7A7, EEF1A2, MRC1, NFKBIE, CPNE5, PLEKHO1, IFIH1, SMO, RARRES3, SAMSN1, GNAS, MAP3K3, TMEM63B, OAS2, VCAM1, LRP5, HERC5, VMO1, TSPAN33, CCR1, ARC, AOAH, HSPA6, CXCR4, TCIRG1, LY86, BLOC1S2, GGA2, GSTA4, COPS7A, RCSD1, MMP15, PSMB1, SOCS3, STAT2, SH3BP1, EVI2A, SERPINB6, GALM, RPS6KA1, ALDH7A1, FAM19A5, RNF122, IL7R, GRK6, CCT3, PPFIA1, ACSL5, RARRES2, AGPAT1, PLEK, SCPEP1, RHBDF2, EVL, CD8B, CTLA4, IFIT2, GAL, PSKH1, TNFSF13B, HLA-DMB, ZFP36L2, LPXN, GPRC5A, MAP4K1, TLCD1, LYPD1, HSPA1B, SH2D2A, ZNF749, LRRC61, LCP2, TULP3, TUSC1, MYO5B, SHC2, DBN1, HES1, BATF2, ACTB, CD4, SLC6A15, TMEM98, PHF1, TMEM51, SELL, ARHGEF1, GZMK, CECR7, IRF7, DNAJB1, ADAM23, RAPGEF1, TNFSF13, BCHE, HOXB13, GSTK1, SEMA4C, TRAF7, EBI3, CD3D, KIT, MYO1F, EFNB3, PA2G4, MICB, BTG2, PARP12, SIT1, UNC13D, RBP5, BATF, F13A1, CXCL12, CCL2, SELPLG, TP53INP1, DPYSL4, RAB17, AIF1, FAM57A, RHBDF1, IL2RB, PDLIM1, STAT3, MANEAL, CEBPB, SUSD2, RGS14, CTTN, NAB2, CD6, TTLL12, WIPF1, CD38, VANGL2, ELF4, HMOX1, CSF1R, MYH9, CD19, STK10, PLCB2, NPL, CMTM7, GIMAP7, CCL18, LILRB2, LST1, RRS1, CPNE7, IDH2, NUB1, SPN, HLA-DOA, SHKBP1, EPB41, CTSW, AKR1A1, GZMM, EPHX2, POMGNT1, VDAC1, AP1S1, TRIM8, LAMC3, LIPA, GRB2, ELL2, NEO1, ATF5, SIGLEC10, CD163, OASL, MPEG1, PLXNB1, ST6GAL1, FOXRED2, TNFAIP2, LXN, C3AR1, MDH2, ENTPD6, ARHGEF17, OAS3, PLA2G7, VDR, DAZAP2, PDPN, GBP5, HAVCR2, KIAA1755, LRP3, IL2RG, CBX7, ST6GALNAC4, ME1, SLA, NPR2, CD5, DEAF1, HLA-G, DENND2D, TCL1A, HIST2H2BE, CDKN2D, AXL, ALPL, HSF4, SUSD3, PAK1, IFIT3, CD53, ANXA7, CSF2RA, TNFRSF21, IKZF3, BLVRA, PYHIN1, FGR, VASP, CD247, ATP2A3, GNAI2, OPTN, MAN2B1, CETP, IGSF6, GMFG, ERF, NCF4, EPB41L3, CASP1, PGD, MGST1, CD8A, FOXF2, MAPKAPK2, NCF1C, CLIC2, NCF2, BAG3, MAF, TMEM9, RTP4, IL16, DIAPH1 |
|  |  | |  |  |
|  |  | |  |  |
|  |  | |  |  |
|  |  | |  |  |
|  |  | |  |  |
|  |  | |  |  |
|  |  | |  |  |
|  |  | |  |  |
|  |  | |  |  |
|  |  | |  |  |
|  |  | |  |  |
|  |  | |  |  |
|  |  | |  |  |
|  |  | |  |  |
|  |  | |  |  |
