# Supplementary material for: Exploration of the immune cell infiltration-related gene signature in the prognosis of melanoma
Source: Aging (Albany NY). 2021 Jan 10;13(3):3459–82. doi: 10.18632/aging.202279 (PMC7906183; doi:10.18632/aging.202279)
Supplement: Supplementary Table 3 [file aging-13-202279-s004.pdf]

SUPPLEMENTARY TABLE

Supplementary Table 3. Multivariate Cox regression analysis.

ID	Coef	HR	HR.95L	HR.95H	p-Value
CYTL1	0.007408	1.007435	1.002858	1.012033	0.00143
CCL8	-0.033	0.967543	0.929431	1.007217	0.107561
FCGR2C	-0.02117	0.979052	0.960903	0.997543	0.026581
OAS1	-0.0147	0.985407	0.968316	1.0028	0.099617
HAPLN3	-0.03669	0.963978	0.937684	0.991009	0.009321
WIPF1	-0.01375	0.98634	0.975503	0.997297	0.01468
CLIC2	-0.02314	0.97713	0.949366	1.005705	0.115682

Coef: Coefficient; HR: hazard ratio.
